# CPA-seq reveals small ncRNAs with methylated nucleosides and diverse termini

**DOI:** 10.1038/s41421-021-00265-2

**Published:** 2021-04-19

**Authors:** Heming Wang, Rong Huang, Ling Li, Junjin Zhu, Zhihong Li, Chao Peng, Xuran Zhuang, Haifan Lin, Shuo Shi, Pengyu Huang

**Affiliations:** 1 School of Life Science and Technology, ShanghaiTech University, Shanghai, 201210 China; 2 University of Chinese Academy of Sciences, Beijing, 100049 China; 3 CAS Center for Excellence in Molecular Cell Science, Chinese Academy of Sciences, Shanghai, 200031 China; 4 National Facility for Protein Science in Shanghai, Zhangjiang Lab, Shanghai Advanced Research Institute, Chinese Academy of Science, Shanghai, 201210 China; 5 Yale Stem Cell Center and Department of Cell Biology, Yale University School of Medicine, New Haven, CT 06520 USA; 6 Shanghai Institute for Advanced Immunochemical Studies (SIAIS), ShanghaiTech University, Shanghai, 201210 China; 7 Institute of Biomedical Engineering, Chinese Academy of Medical Sciences and Peking Union Medical College, Tianjin, 300192 China

**Keywords:** Gene expression profiling, Transcriptomics

## Abstract

High-throughput sequencing reveals the complex landscape of small noncoding RNAs (sRNAs). However, it is limited by requiring 5′-monophosphate and 3′-hydroxyl in RNAs for adapter ligation and hindered by methylated nucleosides that interfere with reverse transcription. Here we develop Cap-Clip acid pyrophosphatase (Cap-Clip), T4 polynucleotide kinase (PNK) and AlkB/AlkB(D135S)-facilitated small ncRNA sequencing (CPA-seq) to detect and quantify sRNAs with terminus multiplicities and nucleoside methylations. CPA-seq identified a large number of previously undetected sRNAs. Comparison of sRNAs with or without AlkB/AlkB(D135S) treatment reveals nucleoside methylations on sRNAs. Using CPA-seq, we profiled the sRNA transcriptomes (sRNomes) of nine mouse tissues and reported the extensive tissue-specific differences of sRNAs. We also observed the transition of sRNomes during hepatic reprogramming. Knockdown of mesenchymal stem cell-enriched U1-5′ snsRNA promoted hepatic reprogramming. CPA-seq is a powerful tool with high sensitivity and specificity for profiling sRNAs with methylated nucleosides and diverse termini.

## Introduction

Small noncoding RNAs (sRNAs) of 15–40 nucleotides in length comprise a large family of microRNA (miRNA), small interfering RNA (siRNA), PIWI-interacting RNA (piRNA), as well as tsRNA, rsRNA, snsRNA, snosRNA, and lncsRNA that are processed from tRNA, ribosomal RNA (rRNA), small nuclear RNA (snRNA), small nucleolar RNA (snoRNA) and long noncoding RNA (lncRNA), respectively^1–3^. sRNA profiling by high-throughput sRNA sequencing (sRNA-seq) provides insights into the intricate landscape of sRNAs^4^. To generate sRNA libraries for high-throughput sequencing, sRNA molecules are usually ligated to 3′ and 5′ adapters followed by reverse transcription and PCR amplification^5^. This prevailing sRNA-seq method has been widely used for quantitative studies of miRNAs and piRNAs, as well as other sRNAs. However, methylated nucleosides, which are abundant in tsRNA, often result in pauses, stops, or misincorporations during reverse transcription^6–8^. Moreover, the commonly used sRNA-seq methods usually require 5′-monophosphate (5′-P) and 3′-hydroxyl (3′-OH) in the RNA molecule for adapter ligation^9^. Recently, there have been increasing reports showing the existence of 5′-hydroxyl (5′-OH), 5′-cap, 5′-triphosphate (5′-ppp), 3′-phosphate (3′-P), 2′,3′-cyclic phosphate (3′-cP), and 3′-aminoacyl (3′-aa) in eukaryotic sRNAs, which hampered adapter ligation^10–12^. Thus, significant sub-populations of sRNAs are not detected by commonly used sRNA-seq methods; these sRNAs may form a hidden layer of the transcriptome.

Several strategies have been developed to overcome the obstacles of sequencing RNAs with terminus multiplicities and nucleoside methylations. Alkali treatment was used to remove aminoacyl residues from charged tRNAs^13^. Multiple decapping enzymes were used to hydrolyze the phosphoric acid anhydride bonds in the triphosphate bridge of the cap structure to generate 5′-P termini for 5′ adapter ligation, including for example tobacco acid pyrophosphatase (TAP), RNA 5′ pyrophosphatase (RppH), and Cap-Clip acid pyrophosphatase (Cap-Clip), all of which are capable of decapping both 7-methylguanosine (m^7^G) and 2,2,7-trimethylguanosine (m^3^G) caps^14–18^. Moreover, pyrophosphatases are also capable of generating 5′-P termini from 5′-triphosphorylated RNA^19^.

Another enzyme used to reduce terminus multiplicity is T4 polynucleotide kinase (PNK), which catalyzes the phosphorylation of 5′-OH termini to generate 5′-P and removal of phosphoryl groups from 3′-P and 2′,3′-cyclic phosphate (3′-cP) termini to generate 3′-OH^20,21^. Dicer and Drosha each generate 5′-P and 3′-OH termini in miRNAs; several other ribonucleases, such as Angiogenin, produce 5′-OH, 3′-P, or 3′-cP termini in other sRNA types^10,22,23^. Thus, T4 PNK has been employed for sequencing of tsRNA^24^ and cyclic phosphate-containing-RNAs^25^, as well as circulating lncsRNA and mRNA-derived sRNAs^26^.

To overcome the obstacles in reading through methylation sites during reverse transcription, two strategies have been developed. One strategy is to pre-treat RNA with demethylase. In previous studies, AlkB has been reported to efficiently remove methylations in *N*^1^-methyladenosine (m^1^A), *N*^3^-methylcytosine (m^3^C), and the AlkB(D135S) variant can efficiently demethylate *N*^1^-methylguanosine (m^1^G); both of these have been used to facilitate sequencing of tRNAs and their derivants^27,28^. Another strategy is to use reverse transcriptases with high processivity in reverse transcription of highly structured or heavily modified RNAs, such as thermostable group II intron reverse transcriptase (TGIRT) and an evolved form of the HIV-1 reverse transcriptase, both of which can introduce misincorporation at methylation sites^29–31^.

Using Cap-Clip, T4 PNK, and AlkB/AlkB(D135S)-facilitated small ncRNA sequencing (CPA-seq), we profiled the sRNome of human embryonic kidney cells (HEK293T), and revealed sRNAs with terminus multiplicities and nucleoside methylations. Comparing sRNA with or without treatment of AlkB mix, we estimated the methylation status of tsRNAs. We also profiled the sRNA transcriptomes (sRNomes) of nine mouse tissues. CPA-seq revealed similar tissue-specific expression patterns of miRNAs as in previous reports^3,32–34^. However, compared to previously reported sRNA atlases across different mouse tissues generated by the conventional sRNA-seq methods, we observed more complex sRNA profiles across mouse tissues^32–34^. We found that a large number of tsRNAs, snsRNAs, snosRNAs, and lncsRNAs also showed tissue-specific expression patterns. The expression patterns of sRNAs in specific cell types could be remodeled upon cell fate conversion. Thus, the sRNomes generated using CPA-seq in this study could facilitate studies of sRNAs in mammalian tissues.

## Results

### Overview of CPA-seq

**Fig. 1:**
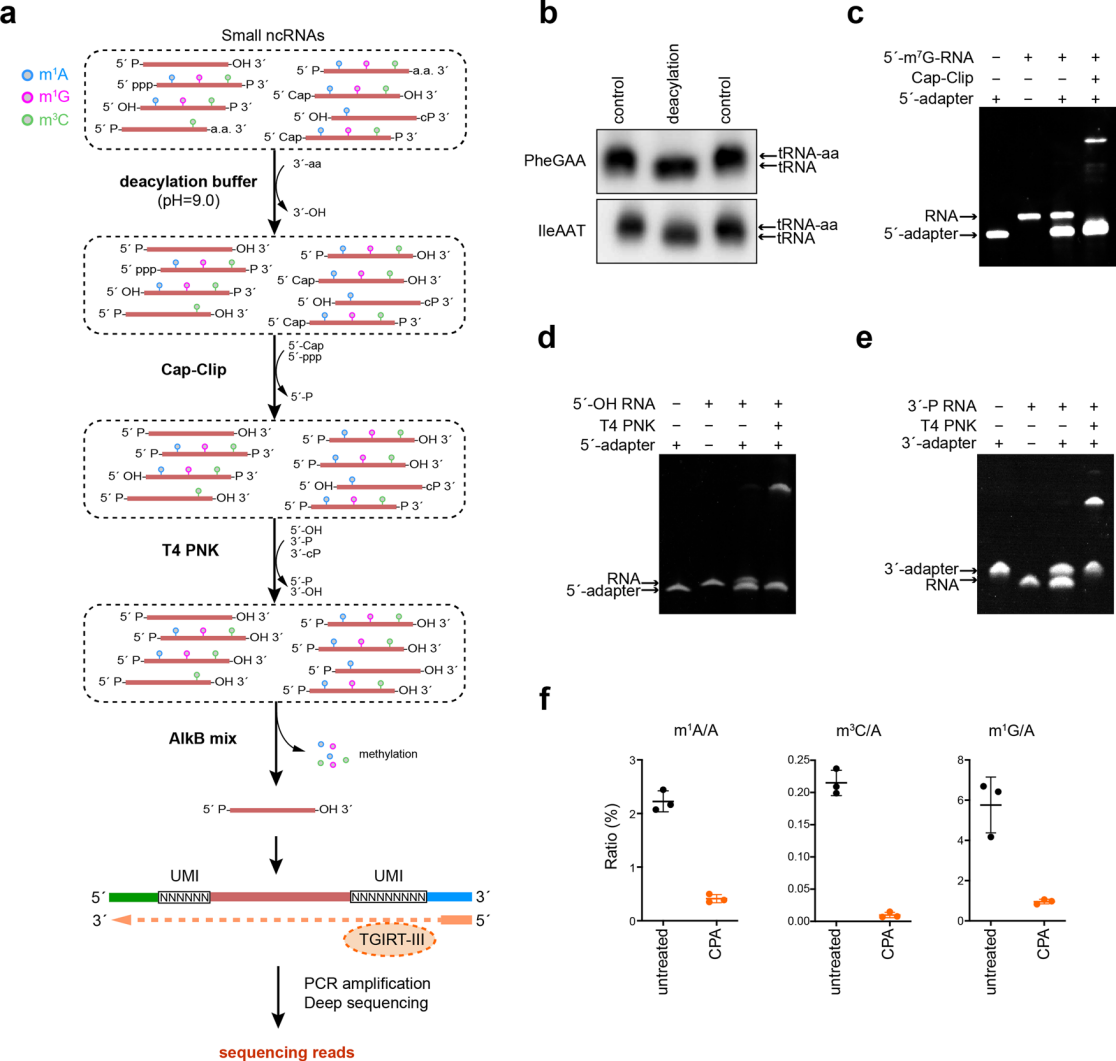
CPA-seq. **a** The workflow of sRNA library preparation for CPA-seq. Purified small RNAs are incubated in deacylation buffer to remove 3′-aminoacyl (3′-aa), treated with Cap-Clip to remove 5′ m^7^G and m^3^G caps, then treated with T4 PNK to convert 5′-OH to 5′-P, and to convert 3′-P and 3′-cP to 3′-OH, followed by treatment with a mix of AlkB and AlkB(D135S) to remove methylations in m^1^G, m^3^C, and m^1^A. The pretreated small RNAs were ligated with 3′ and 5′ adapters, reverse transcribed by TGIRT-III, and then PCR amplified for sequencing. **b** Northern blotting of RNA samples from HEK293T with/without treatment of deacylation buffer. **c** Cap-Clip treated synthetic 5′-m^7^G-RNA (31 nt) was ligated with a 5′-adapter (26 nt). **d** T4 PNK-treated synthetic 5′-OH RNA (27 nt) was ligated with a 5′-adapter (26 nt). **e** T4 PNK-treated synthetic 3′-P RNA (27 nt) was ligated with 3′-adapter (29 nt). **f** LC-MS/MS analysis showed that sequential treatments with deacylation buffer, Cap-Clip, T4 PNK, and AlkB mix (CPA) efficiently removed methylations in m^1^G, m^3^C, and m^1^A of small RNAs extracted from HEK293T cells (*n* = 3).

Here, we developed CPA-seq to overcome common obstacles described above that impede preparation of sRNA libraries (Fig. 1a). First, we incubated sRNAs in deacylation buffer (pH = 9.0) to remove aminoacyl residues in aminoacyl-tRNA-derived 3′-tsRNAs (Fig. 1b and Supplementary Fig. S1a)^13^. Second, we used Cap-Clip to remove the 5′-cap and 5′-ppp from RNAs to generate 5′-P termini. We compared two commercially available decapping enzymes, RppH and Cap-Clip. In our hands, Cap-Clip was superior to RppH for preserving RNA integrity (Supplementary Fig. S1b). Cap-Clip efficiently removed the 5′-m^7^G cap from a synthetic 5′-m^7^G-capped short RNA to enable 5′-adapter liagation, showing high decapping efficiency^15,35^ (Fig. 1c and Supplementary Fig. S1c).

Third, we used T4 PNK to reduce terminus multiplicities (Fig. 1d, e and Supplementary Fig. S1d, e). T4 PNK efficiently phosphorylated 5′-OH and removed phosphoryl groups from 3′-termini of synthetic RNA oligos bearing 5′-OH and 3′-P, thereby enabling efficient adapter ligation (Fig. 1d, e and Supplementary Fig. S1d). Fourth, a mixture of AlkB and AlkB(D135S) (AlkB mix) was used to remove methylations in m^1^A, m^3^C, and m^1^G. We optimized the AlkB mix reaction for demethylation efficiency while retaining RNA integrity (Fig. 1f and Supplementary Fig. S1f–k). After sequential deacylation and CPA treatments, sRNAs were ligated to 5′ and 3′ degenerate adapters containing unique molecular identifiers (UMIs)^36^, reverse transcribed by TGIRT-III, and followed by PCR amplification (Fig. 1a).

We tested the sensitivity of CPA-seq. CPA-seq of 25–100 ng of small RNA extracted from HEK293T cells revealed comparable species numbers of different sRNA types (Supplementary Fig. S1l–n).

### Performance comparisons among CPA-seq and commercial sRNA-seq methods

To evaluate the performance of CPA-seq, we sequenced sRNAs from HEK293T cells using CPA-seq and using three commercially available library preparation methods for sRNA-seq (NEBNext, QIAseq, and TruSeq, collective shortened to “NQT-seq”; Supplementary Fig. S2a). Note that among the NQT-seq methods, QIAseq uses UMI-containing RT primer to reduce bias.

Sequencing reads corresponding to sRNAs of 16–40 nucleotides (nt) in length were used for subsequent analyses. The sequencing reads were aligned sequentially to known miRNA, rRNA, cytosolic tRNA, piRNA, mitochondrial tRNA, lncRNA, snRNA, snoRNA, and other ncRNA types (“Materials and methods” and Supplementary Fig. S3).

The distributions of sRNA types varied using different sRNA library preparation methods (Supplementary Fig. S2b, Table S1). MiRNAs are known to contain extensive 5′-P and 3′-OH termini, and can be captured by all of the tested sRNA-seq methods. We observed that the miRNA species detected with all of the tested methods shared large overlap (Supplementary Fig. S2c). However, CPA-seq revealed much more species of tsRNA, lncsRNA, snsRNA, snosRNA, rsRNA, mRNA-derived, and other ncRNA-derived sRNAs, suggesting the terminus multiplicities and nucleoside methylation in non-miRNA sRNAs (Supplementary Fig. S2c).

We performed Northern blotting to verify the performance of different library preparation methods for the detection of non-miRNA sRNAs. The sRNA profile detected by CPA-seq matched the northern blotting banding pattern more closely than the profiles generated using the NQT-seq methods (Supplementary Fig. S4).

### CPA-seq reveals sRNAs with diverse termini

**Fig. 2:**
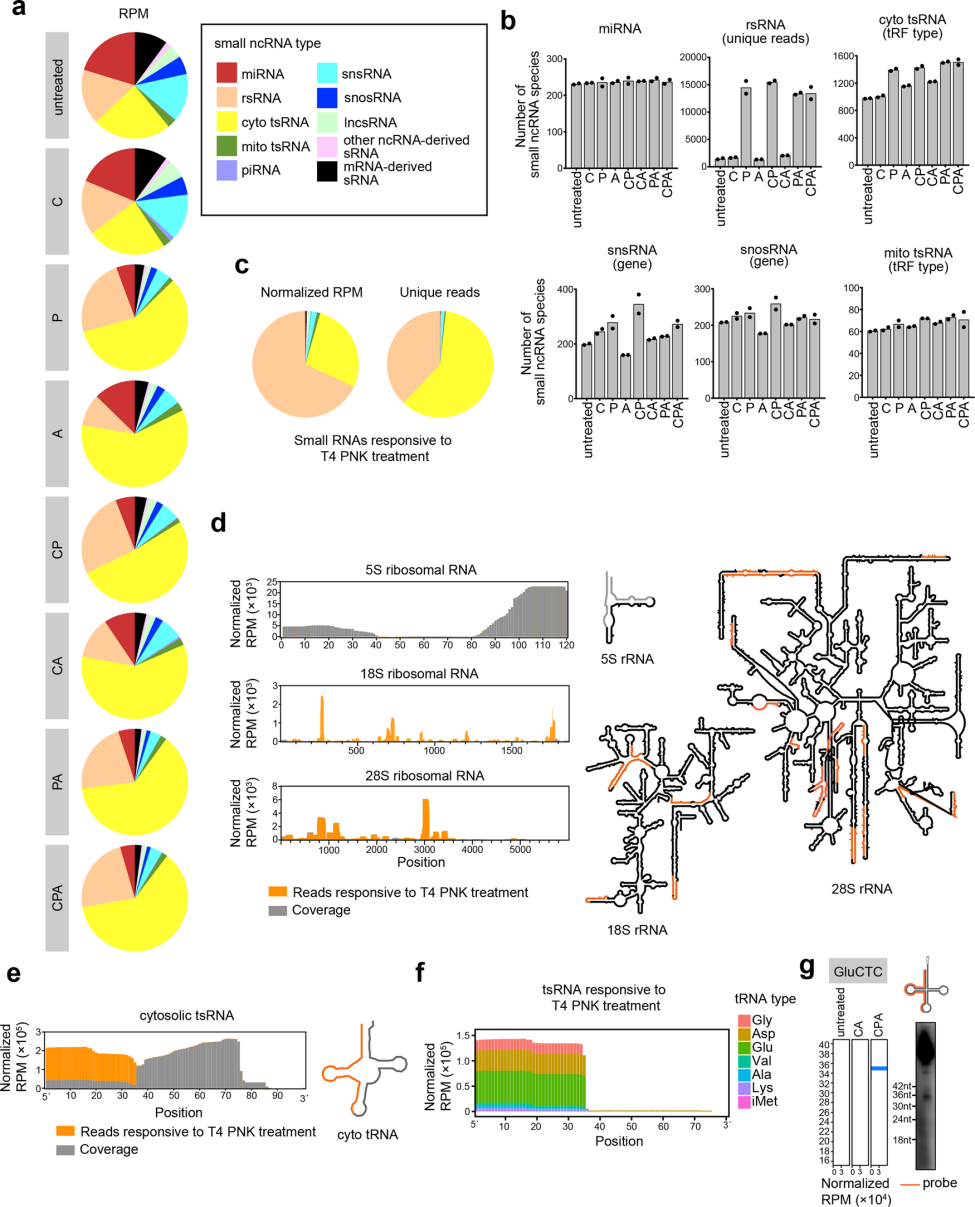
CPA-seq reveals sRNAs with diverse termini. **a** Distribution of different types of sRNAs extracted from HEK293T cells that we process with the full CPA-seq process or with various combinations of the Cap-Clip, T4 PNK, and AlkB mix enzymes (*n* = 2). **b** The number of sRNA species revealed with different treatments (*n* = 2). **c** Distribution of different types of sRNAs responsive to T4 PNK treatment (unique reads that were highly detected in CPA group, but lowly detected in CA group with the fold change > 30, *n* = 2). **d** Reads of sRNA responsive to T4 PNK treatment mapping to 5S, 18S, and 28S ribosomal RNAs have been combined to show detection of rsRNAs containing diverse termini (rsRNAs with RPM > 300 are shown in the structural map). **e** Reads of sRNA responsive to T4 PNK treatment mapping to cytosolic tsRNAs have been combined to show detection of tsRNAs containing diverse termini. **f** Reads of tsRNAs responsive to T4 PNK treatment. **g** Northern blotting of GluCTC 5′tsRNAs that are responsive to T4 PNK treatment.

Next, we performed sRNA sequencing of small RNA extracted from HEK293T cells that we process with the full CPA-seq process or with various combinations of the Cap-Clip, T4 PNK, and AlkB mix enzymes. Unsurprisingly, distinct distributions of the various sRNA types were detected upon these different treatments (Fig. 2a, b and Supplementary Table S1). The miRNAs revealed by different treatments showed a high correlation (Supplementary Fig. S5a), suggesting the low terminus multiplicity of the miRNAs. Thus, we normalized the RPM value to total miRNA RPM in the following analyses to estimate the amount of different sRNA species revealed by sRNA-seq with different treatments.

We first compared the sRNAs detected in CA and CPA groups to analyze the sRNAs responsive to T4 PNK treatment, which putatively contain 5′-OH, 3′-P, or 3′-cP. A majority of the sRNAs responsive to T4 PNK treatment (unique reads that were highly detected in CPA group, but lowly detected in CA group with the fold change >30) were found to be rsRNAs and tsRNAs (Fig. 2b, c).

Surprisingly, we found that the majority of the rsRNAs that could be captured without T4 PNK treatment were derived from 5S ribosomal RNA. On the contrary, the majority of the 18S and 28S ribosomal RNA-derived rsRNAs require T4 PNK treatment to be captured by sRNA-seq, and putatively contain 5′-OH, 3′-P, or 3′-cP termini (Fig. 2d). The 18S and 28S ribosomal RNA-derived rsRNAs are preferentially generated by the cleavages at bubble region of the RNA (Fig. 2d). The distinct 5′ or 3′ termini of 5S and 18S/28S rRNA-derived sRNAs suggested different mechanisms for generation of these rsRNAs.

tsRNAs that are responsive to T4 PNK treatment are mapped mainly to 5′ parts of tRNAs (Fig. 2e–g). The T4 PNK-responsive tsRNAs are mostly generated by the cleavage at the anticodon loop of the tRNA (Fig. 2e). Previous studies have reported Angiogenin, a stress-activated endonuclease, cleaves tRNAs within the anticodon loop to generate 5′ tsRNA under stress^10,23^. The Angiogenin is also known to generate 5′-P and 3′-cP, which hamper the ligation of adapters to tsRNAs generated by Angionenin^37^. However, the large amount of T4 PNK-responsive 5′ tsRNA from HEK293T cells without stress cannot be simply explained by Angiogenin-mediated biogenesis of tsRNA. Whether other mechanisms are involved in the biogenesis of tsRNAs with 5′-OH, 3′-P, and 3′-cP termini should be investigated in the future.

### CPA-seq reveals 5′-capped sRNAs

**Fig. 3:**
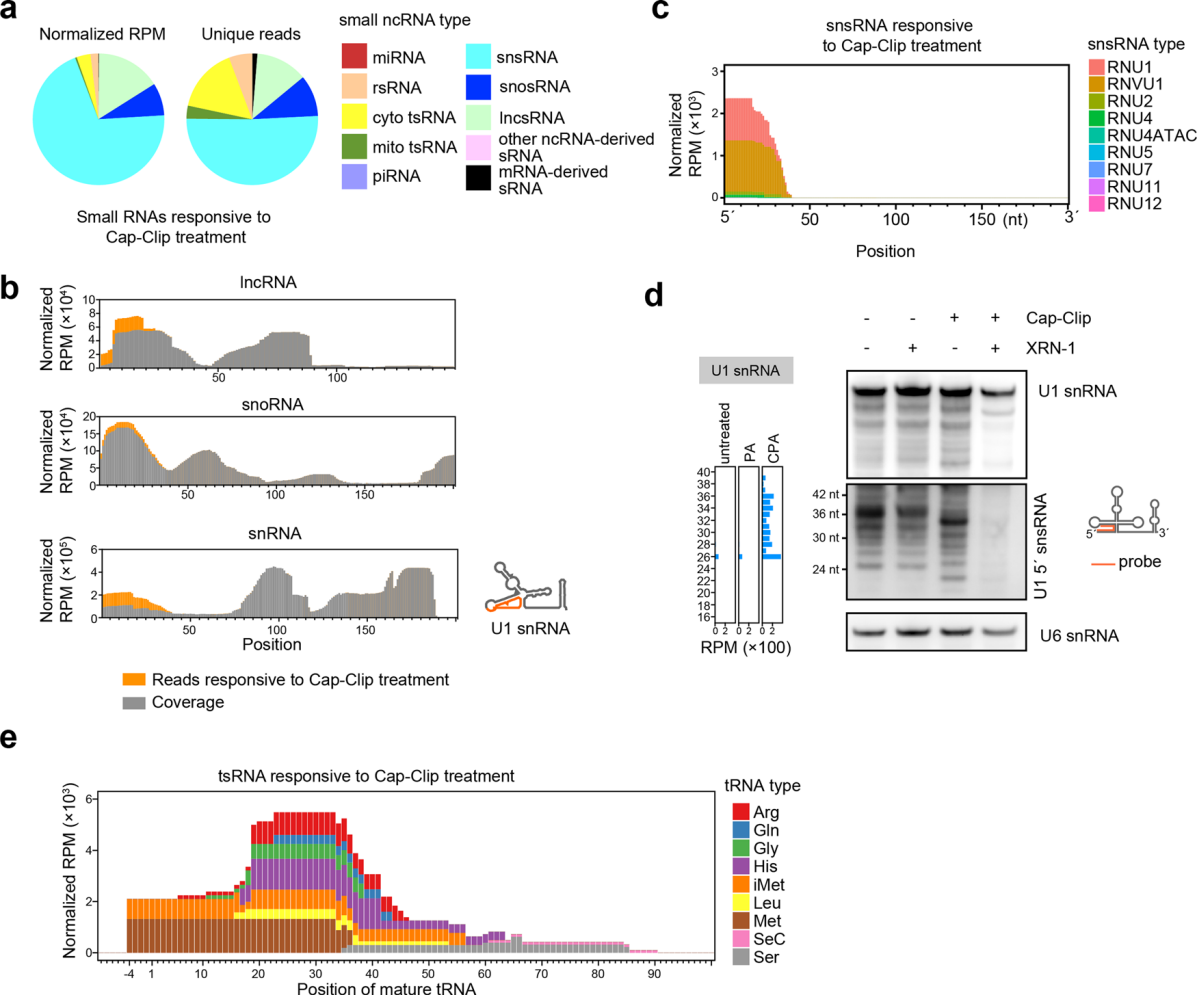
CPA-seq reveals sRNAs with 5′-cap and 5′-ppp. **a** Distribution of different types of sRNAs responsive to Cap-Clip treatment (unique reads that were highly detected in CPA group, but lowly detected in PA group with the fold change >30, *n* = 2). **b** Reads of sRNA responsive to Cap-Clip treatment mapping to lncRNAs, snoRNAs, and snRNAs have been combined to show the detection of lncsRNAs, snosRNAs, and snsRNAs putatively containing 5′-caps or 5′-ppp. **c** Reads of snsRNAs responsive to Cap-Clip treatments. **d** Northern blotting of sRNAs treated with the indicated enzymes, using a probe complementary to U1-5′snsRNA. Cap-Clip removed the 5′-cap from U1-5′snsRNAs and enabled U1-5′snsRNAs to be digested by XRN-1 (lane 4), a 5′→3′ exoribonuclease that requires 5′-P. Removal of the 5′-cap by Cap-Clip leads to a slight shift of the bands representing U1-5′snsRNAs (lane 3). U6 snRNA is used as a reference. **e** Reads of tsRNAs responsive to Cap-Clip treatments.

Next, we compared the sRNAs detected in the PA and CPA group to analyze the sRNAs responsive to Cap-Clip treatments; these were expected to include RNAs contain a 5′-cap or 5′-ppp. In consistent with a previous study using TAP to sequence 5′-capped sRNAs^16^, we also observed many snsRNAs, lncsRNAs, and snosRNAs that were responsive to Cap-Clip treatment (Fig. 3a). We found that the lncsRNA, snosRNA, and snsRNA reads mapped mainly to 5′ parts of their corresponding full-length RNAs, which usually contain 5′-caps or 5′-ppp (Fig. 3b). Cap-clip responsive snsRNAs were mainly derived from Sm-class snRNAs, including RNU1 and RNVU1, which are well-characterized to be 5′-capped with m^3^G^38^ (Fig. 3c). Using a probe recognizing the 5′ parts of U1 snRNA, we found that the Cap-Clip-decapped U1 snRNAs and their 5′ snsRNAs (U1-5′ snsRNAs) ran slightly faster in electrophoresis and were readily digested by XRN-1, a 5′→3′ exoribonuclease requiring 5′-P (Fig. 3d). This result validated the existence of 5′-capped U1-5′ snsRNAs in HEK293T cells.

We surprisingly observed a few Cap-Clip responsive tsRNAs with 5′ cleavage sites within the 5′-leader sequence of tRNA precursor or 5′ parts of mature tRNA (Fig. 3e). As these Cap-Clip-responsive tsRNAs do not contain the 5′-termini of their corresponding tRNA precursors, they are possibly de novo capped or triphosphorylated.

### CPA-seq reveals methylated sRNAs

**Fig. 4:**
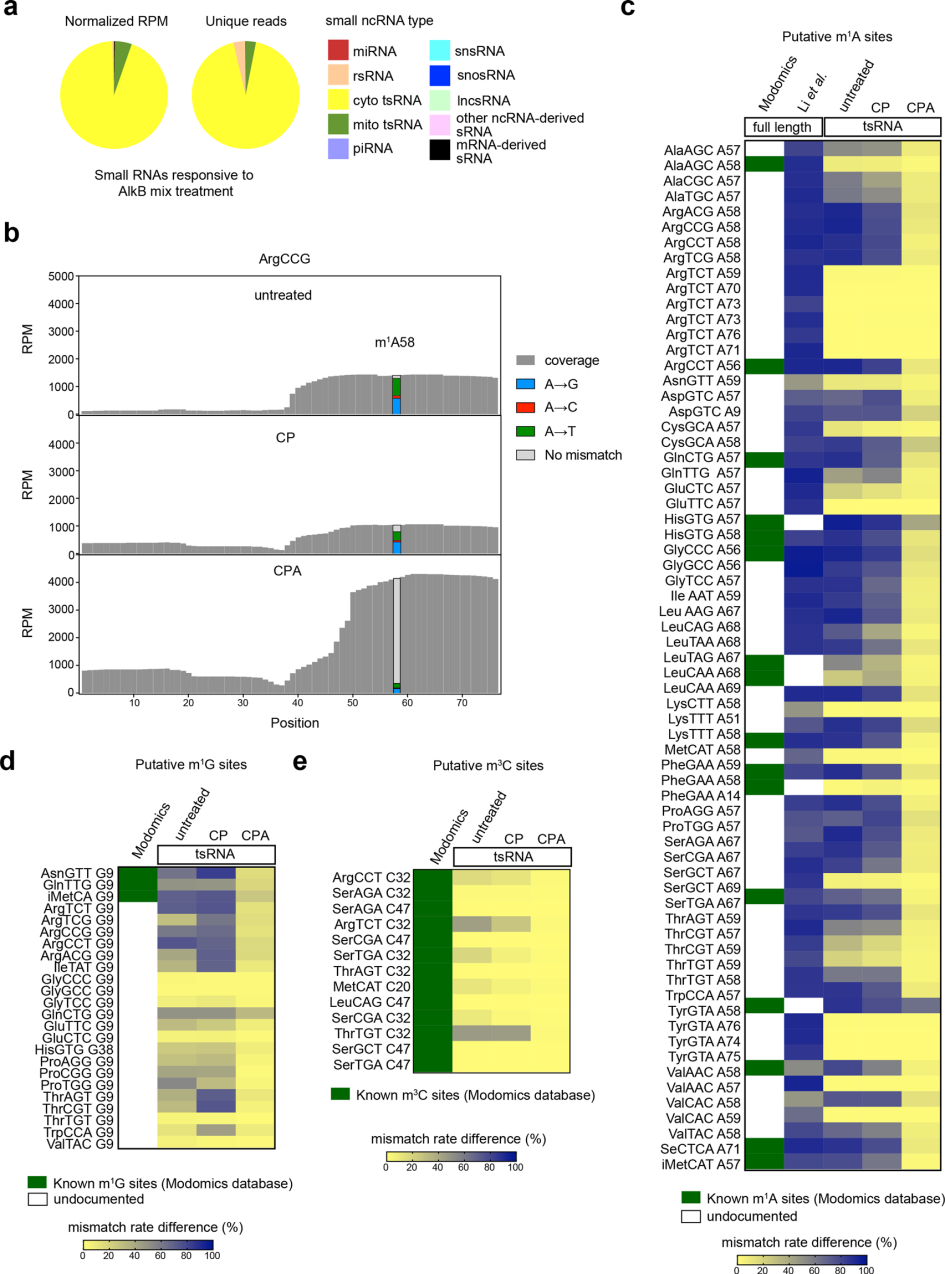
Methylome of sRNAs. **a** Pie diagrams showing the distribution of different types of sRNAs responsive to AlkB mix treatment (unique reads that were highly detected in CPA group, but lowly detected in CP group with the fold change >30, *n* = 2). **b** Misincorporation and coverage plot for putative m^1^A sites identified in this study. **c** Estimation of m^1^A methylome by the mismatch frequency at A sites of tRNAs. Treatment of AlkB mix demethylates m^1^A and reduces TGIRT-induced misincorporation at m^1^A sites. The known m^1^A sites (Modomics database) and putative m^1^A sites of full-length tRNAs revealed by Li et al.^40^ are shown accompanied by the m^1^A site of tsRNAs revealed by this study. **d** The m^1^G sites of tsRNAs revealed by CP-seq and CPA-seq. **e** The m^3^C sites of tsRNAs revealed by CP-seq and CPA-seq.

The commonly used reverse transcriptases tend to stop at m^1^A, m^3^C, and m^1^G sites. Thus, it is difficult to sequence sRNAs derived from tRNAs and rRNAs containing m^1^A, m^3^C, and m^1^G sites^28^ (Fig. 4a). Our CPA-seq method uses TGIRT-III, a highly processive reverse transcriptase that has been shown to significantly increase the detection of sRNAs derived from tRNAs containing m^1^A, m^3^C, and m^1^G sites^8,29,39^ (Figs. 2a, 4b). During reverse transcription, TGIRT-III tends to introduce misincorporations and stops at m^1^A, m^3^C, and m^1^G sites, providing us an opportunity to estimate m^1^A, m^3^C, and m^1^G stoichiometries at individual sites across the sRNome.

We compared the sRNAs detected in CP and CPA groups to analyze the sRNAs responsive to AlkB mix treatment, which we expected to include RNAs containing m^1^A, m^3^C, or m^1^G sites. The majority of the sRNAs responsive to AlkB mix treatment are derived from tRNAs (Fig. 4a). We also compared the extent of misincorporation events in the “untreated”, CP, and CPA groups. The significantly reduced number of misincorporation events at putative m^1^A, m^3^C, and m^1^G sites in the CPA group confirmed the high demethylation efficiency of AlkB mix treatment (Fig. 4b–e). We compared the putative m^1^A sites in tsRNAs with known m^1^A sites (Modomics database) and putative m^1^A sites of their corresponding full-length tRNAs revealed by a previous study using the same TGIRT reverse transcriptase as we used in CPA-seq^40,41^. All of the putative m^1^A, m^1^G, and m^3^C sites on tsRNAs revealed in this study were either known m^1^A sites or putative m^1^A sites of their corresponding full-length tRNAs revealed by Li et al.^40^ (Fig. 4c–e and Supplementary Fig. S5b–f). However, we also observed a few putative m^1^A sites revealed by Li et al.^40^ were not methylated at the corresponding sites of tsRNAs, suggesting lower m^1^A methylation frequency of tsRNAs (Fig. 4c).

### Profiling sRNomes of mouse tissues by CPA-seq

We used CPA-seq to profile the sRNomes of nine mouse tissues including testis, stomach, ovary, muscle, lung, liver, kidney, heart, and brain (from 9- to 10-week-old C57BL/6J mice). The sRNA library for each tissue contained between 4.42 and 13.07 million unique reads (Supplementary Table S2, Fig. S6a).

**Fig. 5:**
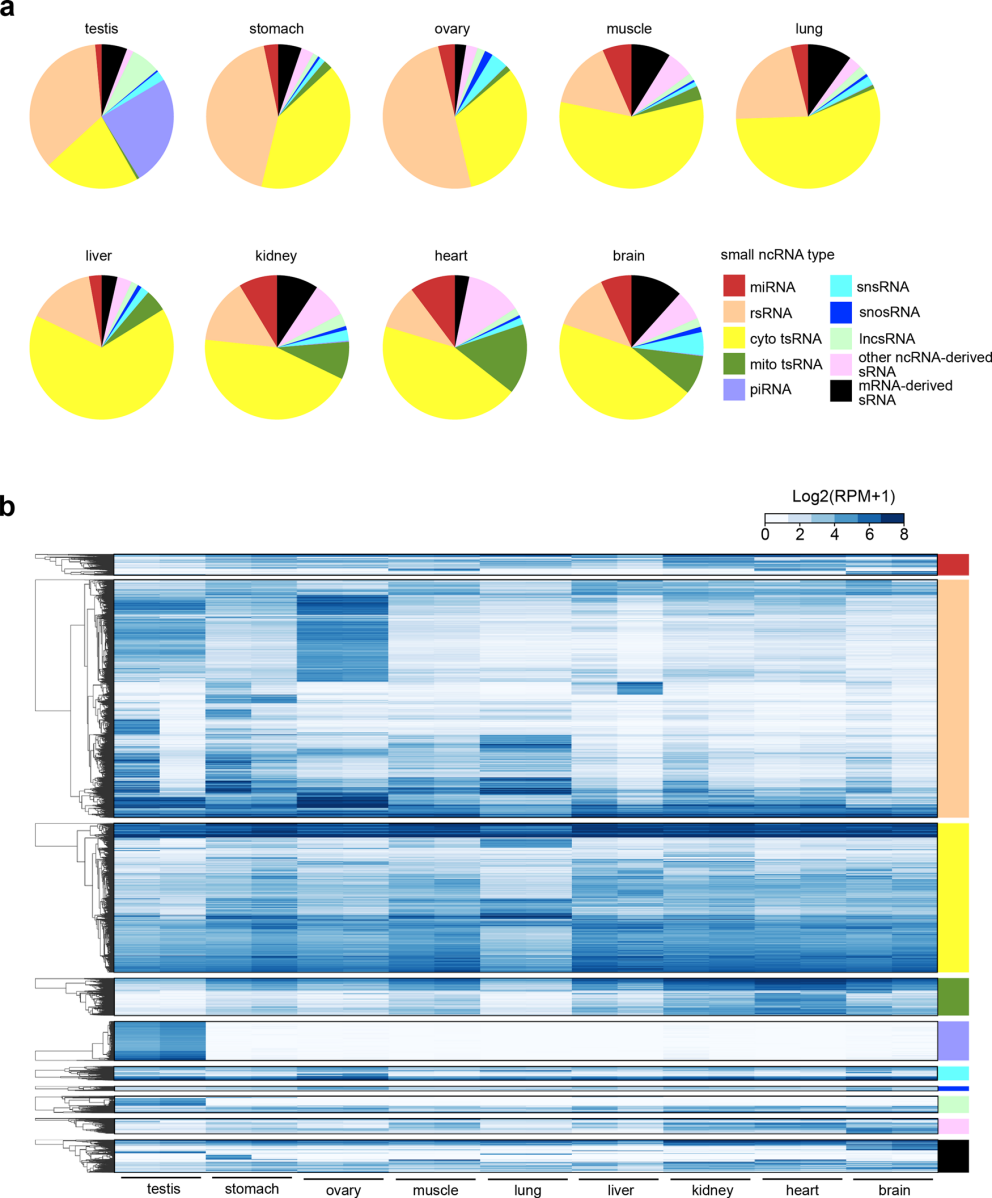
sRNomes across different mouse tissues. **a** Coverage of sRNA types across different mouse tissues as profiled using CPA-seq. **b** Hierarchical clustering of unique reads (RPM > 20 in at least one tissue) from the indicated mouse tissues. For hierarchically-clustered analysis, the unique reads were first classified according to the sRNA type. Then the average Euclidean distance for log_2_ transformed unique reads of each type of sRNA was computed.

We then mapped the sequencing reads to different sRNA types. In total, miRNA contributed to 1.83%–12.32% of all the detected CPA-seq RNA reads in mouse tissues, a small proportion mirroring our findings from the HEK293T cells. rsRNA and tsRNA were the most prominent sRNA types among all mouse tissues profiled with CPA-seq. The relative abundance of other sRNA types varied in different tissues. For example, mitochondrial tsRNAs were abundant in the heart, and energy-demanding organ known to be rich in mitochondria (Fig. 5a). snsRNA and mRNA-derived sRNA were more abundant in the brain (Fig. 5a).

Next, we determined the overall sRNA expression patterns across different tissues. Hierarchical cluster analysis of unique reads of different sRNA types showed that a large number of sRNAs were differentially expressed in different mouse tissues (Fig. 5b). In the *t*-distributed stochastic neighbor embedding (*t*-SNE) projection plot, sRNAs were separated according to their tissue type, representing distinct sRNA expression patterns across various tissues (Supplementary Fig. S6b).

### Tissue-enriched sRNAs

To identify tissue-enriched sRNAs, we calculated the tissue specificity index (TSI) for each detected sRNA using a previously described method^42^. miRNAs are the most extensively studied sRNA type, and our CPA-seq method successfully identified many well-described tissue-enriched miRNAs, including for example the liver-enriched miRNA (miR-122), the heart-enriched miRNA (miR-208a), the testis-enriched miRNA (miR-34b and miR-34c), and several brain-enriched miRNAs (miR-9, miR-124, and miR-128) (Supplementary Fig. S6c)^32–34^. The well-matched tissue-specific miRNA expression patterns between the sequencing results of CPA-seq and previous reports using conventional sRNA-seq methods suggested relatively low multiplicity of 5′/3′-termini and nucleoside methylations in miRNAs.

**Fig. 6:**
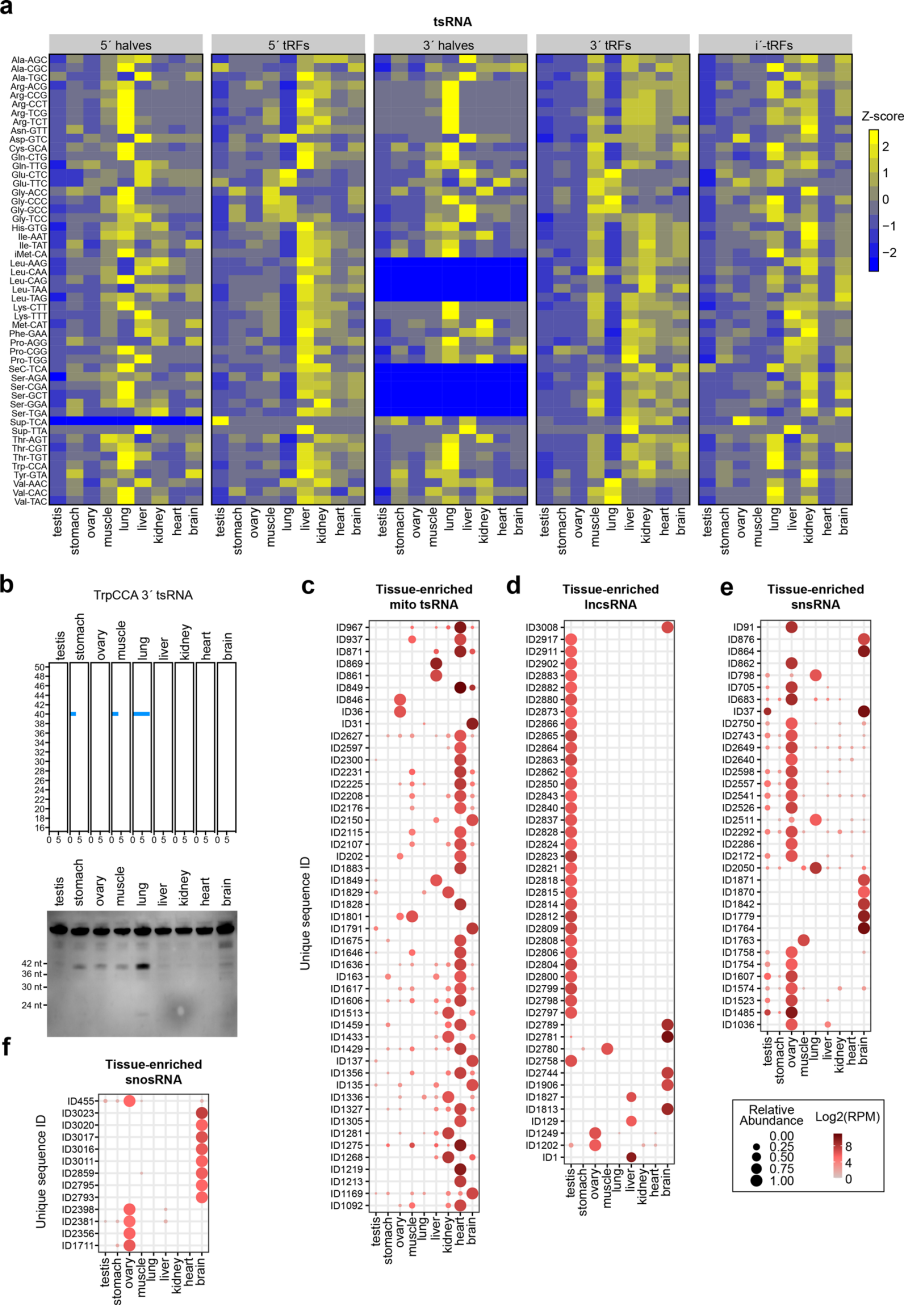
Tissue-enriched sRNAs. **a** Heatmap of the relative abundance of tsRNA\s of different types in the indicated mouse tissues. **b** Detection of lung-enriched TrpCCA 3′tsRNAs by Northern blotting. **c**–**f** Dot plot showing expression patterns of tissue-enriched mitochondrial tsRNAs (TSI > 0.95, RPM > 70 in at least one tissue) (**c**), lncsRNAs (TSI > 0.95, RPM > 50 in at least one tissue) (**d**), snsRNAs (TSI > 0.95, RPM > 50 in at least one tissue) (**e**), and snosRNAs (TSI > 0.95, RPM > 30 in at least one tissue) (**f**).

Recently reports have emphasized the presence and functional impacts of tsRNAs in diverse cell types^43,44^. However, as highlighted by our profiling results above, it is clear that previous surveys conducted with conventional sRNA-seq methods have almost certainly missed very large numbers of tsRNAs, and especially those tsRNAs containing 5′-OH, 3′-P, or methylated nucleosides. We used CPA-seq to systematically analyze the distributions of tsRNAs in mouse tissues. We noted that the lung showed a distinct pattern of tsRNAs (Figs. 5b and 6a, b), which were mainly mapped to 5′-tRNA halves and 3′-tRNA halves (Fig. 6a). Another trend was that the tsRNAs derived from different cytosolic tRNA isodecoders showed distinct tissue-specific expression patterns (Fig. 6a).

Overall, the heart contained the highest numbers of tissue-enriched mitochondrial tsRNAs (Fig. 6c); the testis is enriched for tissue-enriched lncsRNAs (Fig. 6d). Moreover, we observed that the ovary and brain contained the highest numbers of tissue-enriched snsRNAs and snosRNAs among the tested tissues (Fig. 6e, f and Supplementary Table S3).

### Reprogramming of sRNomes during hepatic transdifferentiation

**Fig. 7:**
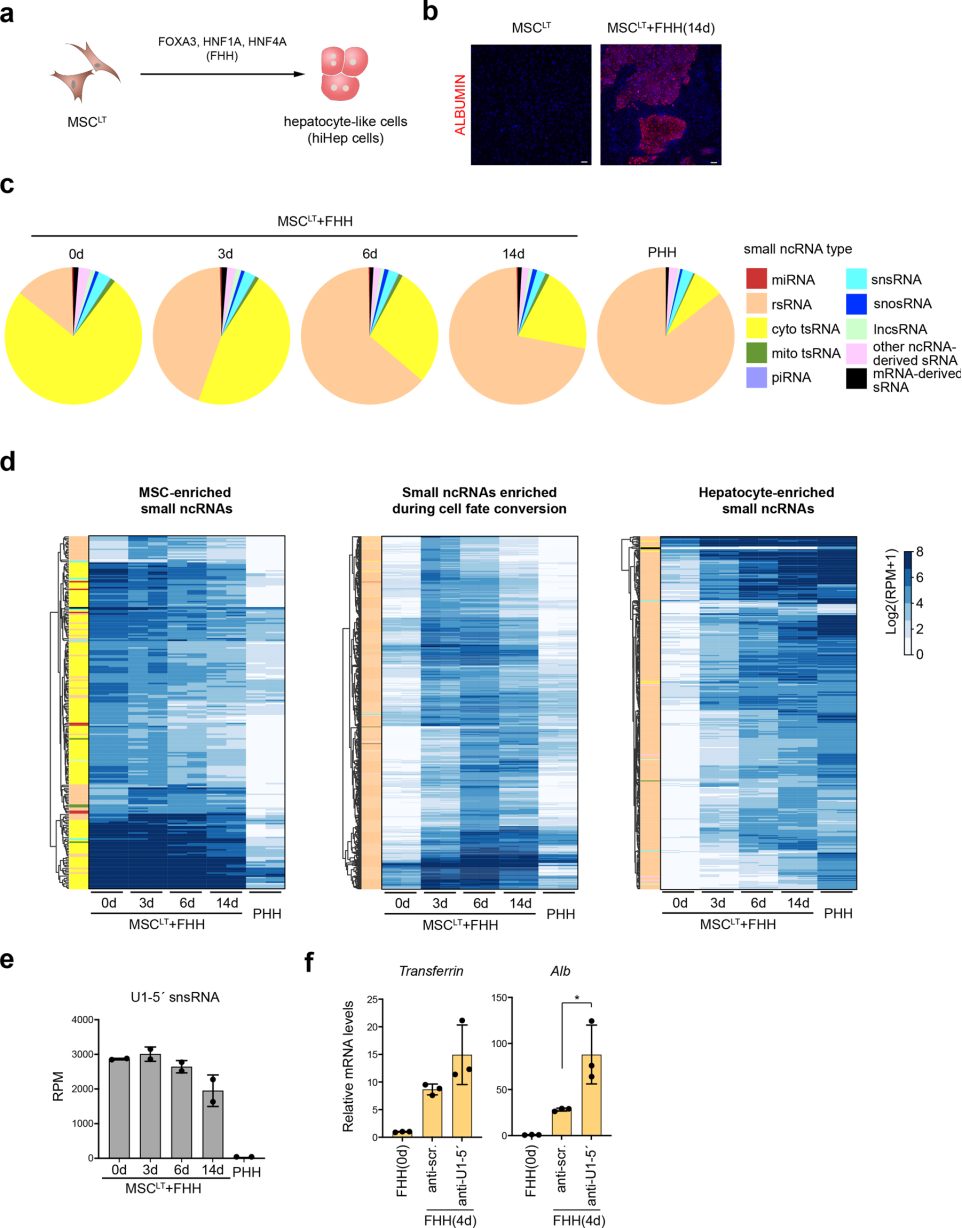
Reprogramming of sRNomes during hepatic reprogramming. **a** Schematic illustration of the strategy used for direct conversion of human MSC^LT^ to hepatocytes. **b** Fluorescent immunostaining of MSC^LT^ and MSC^LT^ infected with *FOXA3*, *HNF1A*, and *HNF4A* (FHH) for 14 days. Scale bar: 100 μm. **c** Distribution of different types of sRNAs in PHH and MSC^LT^ infected with FHH. **d** Hierarchical clustering of unique reads (RPM > 20 in at least one sample) from sequenced samples showing the decreased expressions of MSC^LT^-enriched sRNAs and increased expression of PHH-enriched sRNAs during hepatic reprogramming. **e** Differential expression levels of U1-5′snsRNA in MSC^LT^ and PHH. The expression levels of U1-5′snsRNA gradually decreased in MSC^LT^ cells infected with FHH. **f** Treatment of U1-5′ ASO promoted the induction of hepatic marker genes in MSC^LT^ cells overexpressing FHH.

As many sRNAs showed tissue-specific expression patterns, we questioned whether the sRNome may get reprogrammed as cell fate changes occur. We used a previously described strategy to convert human mesenchymal stem cells expressing SV40 large T antigen (MSC^LT^) into hepatocytes (hiHep cells) by enforced expression of *FOXA3*, *HNF1A*, and *HNF4A* (FHH) (Fig. 7a)^45^. Confirming the success of the conversion, liver cell marker genes were gradually induced in the MSC^LT^ upon FHH expression (Fig. 7b and Supplementary Fig. S7a). We performed CPA-seq on MSC^LT^ undergoing hepatic cell fate conversion and found that the composition of sRNAs transit toward that of primary human hepatocyte (PHH) (Fig. 7c and Supplementary Fig. S7b, c). Supporting the anticipated sRNome reprogramming, we found that FHH expression gradually decreased the abundance of tsRNAs and increased the abundance of rsRNAs (Fig. 7d). Interestingly, the number of rsRNAs increased, while the expression of LeuCAG 3′tsRNA, a ribosomal biogenesis regulator, dramatically decreased upon hepatic reprogramming^43,46^ (Supplementary Fig. S7d).

It was also interesting to observe a significant low-expression level of U1-5′ snsRNA, a 5′-capped sRNA, in primary human hepatocytes (PHH) as compared to that of MSC^LT^ (Fig. 7e). The expression levels of U1-5′ snsRNA in MSC^LT^ gradually decreased after transfection of FHH (Fig. 7e). To investigate the potential role of U1-5′ snsRNA in hepatic reprogramming, we treated MSC^LT^ with an U1-5′ antisense oligonucleotides (ASO), which significantly promoted the induction of hepatic marker genes, suggesting a potentially important role of U1-5′ snsRNA in hepatic reprogramming (Fig. 7f and Supplementary Fig. S7e).

## Discussion

In this study, we developed CPA-seq for profiling sRNAs with terminus multiplicities and nucleoside methylations. This technique enables sensitive identification of a significant fraction of sRNAs that are missed by conventional sRNA-seq methods. These newly detected sRNAs represent a hidden layer of the sRNome.

Recently, many efforts have been made to improve the sRNA sequencing protocols to retrieve previously undetectable sRNA sequences. TGIRT-seq showed higher processivity in RNA-seq but has not been optimized for detection of sRNAs with multiple modifications^29^. Using AlkB, ARM-seq enables sequencing of 3′ tsRNAs containing m^1^A^28^. However, owing to the multiplicity of 5′/3′ termini, ARM-seq is insensitive for sequencing 5′ tsRNAs. T4 PNK has been used to facilitate adapter ligation in preparation of sRNA libraries^47^. However, T4 PNK is not capable of removing the 5′-cap structure, which is known to be abundant in 5′ snsRNAs. Thus, compared to currently available sRNA-seq methods, CPA-seq reveals a more complete view of diverse sRNA species in mammalian cells including but not limited to tsRNAs and snsRNAs. CPA-seq is a powerful tool with high sensitivity for profiling sRNAs that can reveal a more complete picture of the sRNome.

We used CPA-seq to profile the sRNomes of nine mouse tissues and revealed the abundant presence of tsRNA, snsRNA, snosRNA, and lncsRNA, as well as miRNA. These sRNAs showed tissue-specific expression patterns. Accumulating reports are emphasizing the essential biological functions of non-miRNA sRNAs^2^. The sRNomes of different tissues characterized in the present study will likely deepen our understanding of the functional roles of sRNAs in diverse tissues.

Moreover, we gained a deeper insight into the reprogramming of sRNA expression profiles during cell fate conversion by using CPA-seq. The expression of FHH in MSC^LT^ converted MSC^LT^-like sRNome to PHH-like sRNomes. Importantly, we observed a 5′-capped sRNA, U1-5′ snsRNA, functioned as an inhibitor for hepatic reprogramming. Knockdown of U1-5′ snsRNA by U1-5′ ASO can promote hepatic reprogramming.

Although we optimized the sRNA library preparation of CPA-seq, degradation of full-length ncRNA may still contribute to the CPA-seq reads. For example, nucleoside demethylation could lead to the fragility of full-length tRNAs^48^. Using pre-size-selected small RNA for CPA-seq could further eliminate the contamination of full-length RNA degradations.

There are still several types of sRNAs, such as NAD-capped RNA, that cannot be captured by CAP-seq. Improvement of CPA-seq in the future could provide new insight into the compositions of sRNAs.

## Materials and methods

### Molecular cloning and lentivirus production

Plasmids used for expression of *SV40 large T*, *FOXA3*, *HNF1A*, and *HNF4A* were described in previous publication^45^. Constructed plasmids were introduced into HEK293FT cells together with packaging plasmid psPAX2 (Addgene) and envelop plasmid pMD2.G (Addgene). After 48 h incubation, the medium containing lentiviruses was collected and passed through 0.45-μm filter.

### Cell culture and RNA preparation

Human embryonic kidney HEK293T cells and human mesenchymal stem cells were obtained from the American Type Culture Collection (ATCC). HEK293T cells were maintained in DMEM (Thermo) medium supplemented with 10% FBS and 1% 100× penicillin-streptomycin (Gibco) with 5% CO_2_ at 37 °C and human bone marrow-derived mesenchymal stem cells were maintained according to the manufacturer’s instructions. Cryopreserved human hepatocytes from three individuals were provided by Research Institute for Liver Diseases (Shanghai) Co. Ltd and Lonza Walkersville Inc. One donor is a 25-year-old Caucasian male, with no history of smoking and drinking alcohol. The second donor is a 51-year-old Hispanic male with a history of drinking alcohol and no history of smoking. The third donor is a 2-month-old Caucasian boy. Mycoplasma contamination tests were performed routinely. Total or Small RNA was prepared from cells using mirVana miRNA isolation kit (Invitrogen) according to the manufacturer’s instructions. Small RNA used for Northern blotting was purified by RNAiso for (Takara) according to the manufacturer’s instructions.

### Medium

Hepatocyte maintenance medium (HMM) is DMEM/F12 (Gibco) supplemented with 0.544 mg/L ZnCl_2_ (Sinopharm), 0.75 mg/L ZnSO_4_·7H_2_O (Sinopharm), 0.2 mg/L CuSO_4_·5H_2_O (Sinopharm), 0.025 mg/L MnSO_4_ (Sinopharm), 2 g/L Bovine serum albumin (Sigma-Aldrich), 2 g/L Galactose (Sigma-Aldrich), 0.1 g/L Ornithine, 0.03 g/L Proline, 0.61 g/L Nicotinamide, 1× Insulin-transferrin-sodium selenite media supplement (Sigma-Aldrich), 40 ng/mL TGFα (Peprotech), 40 ng/mL EGF (Peprotech), 10 µM dexamethasone, 10 µM Y-27632 (MCE), 0.5 µM A-83-01 (Tocris), 3 µM CHIR99021 (Sigma-Aldrich).

### Animals

Wild-type 9–10-week-old C57BL/6J mice (Charles River Laboratories, China) were anesthetized by intraperitoneal injection of 50 mg/kg pentobarbital sodium and then sacrificed by cervical dislocation. Mice were pinned down onto dissecting tray and the ventral surfaces were sprayed with 70% ethanol. We then opened the chest and abdominal cavity of male mice, and used precooled PBS to wash the residual blood from the heart into the body circulation. The testes, stomachs, muscles, lungs, livers, kidneys, hearts, the whole brains of male mice, and the ovaries of female mice were obtained for RNA extraction. The use and care of animals complied with the guideline of the Biomedical Research Ethics Committee of ShanghaiTech University.

### Tissue handling and RNA extraction

Upon collection, tissue samples were sectioned into smaller pieces and submerged in RNA*later®* Solution (ThermoFisher) for 1 h. Then the tissues were removed from solution and preserved in Lysis/Binding buffer (Life Technologies) until further processing. Small RNA was isolated from 100 mg tissues using mirVana miRNA Isolation Kit according to the manufacturer’s instructions. For primary human hepatocytes (PHH), RNA was extracted from PHH cultured for 48 h in HMM medium. RNA integrity number (RIN) values were used to measure RNA integrity. RIN values of mouse tissue samples were assessed by an Agilent 2100 Bioanalyzer (Agilent Biotechnologies Ltd., USA, Supplementary Table S5).

### Conversion of MSC^LT^ to hepatocytes

To induced hepatic cell fate conversion, 2 × 10^5^ human MSC^LT^ were mixed with lentivirus expressing FOXA3, HNF1A, and HNF4A (MOI = 2 for each virus) and seeded on a collagen I-coated 6-cm dish. Two days later, the medium was changed with HMM medium. The HMM medium was replaced every 2 days. RNA integrity number (RIN) values of samples were assessed by an Agilent 2100 Bioanalyzer (Supplementary Table S5).

### Quantification of ribonucleosides by LC-MS/MS

Hundred and fifty nanograms of RNA was first digested by nuclease P1 (NEB, 1U) in 17 μL 1× P1 digestion buffer containing 25 mM NaCl, 2.5 mM ZnCl_2_ at 42 °C for 2 h. Next, 1 μl FastAP Thermosensitive Alkaline Phosphatase (ThermoFisher) and 2 μl 10× FastAP buffer (ThermoFisher) were added to the reaction and incubated at 37 °C for 2 h. Reactions were added 20 μl acetonitrile for futher detection. After centrifuged at 14,000 rpm for 15 min, the supernatant was aspirated for LC-MS/MS analysis. The LC-MS/MS analysis was performed on Agilent 1290 UPLC (Agilent, USA) coupled to AB Sciex 6500 triple quadrupole mass spectrometer (AB Sciex, USA) with the electrospray ionization (ESI) source. A Thermoscientific Hypersil GOLD aQ column (3 µm, 2.1 × 150 mm) was used for ribonucleosides separation with a flow rate at 0.4 ml/min and column temperature of 35 °C. The mobile phases were comprised of (A) 0.1% formic acid in 100% water and (B) 0.1% formic acid in 100% acetonitrile. The gradient elution was carried out as follows: 0–6 min at 0% B; 6–8 min at 0%–1% B; 8–10 min at 1%–6% B; 10–11 min at 6% B; 11–13 min at 6%–50% B; 13–15 min at 50%–70% B; 15–18 min at 75% B; 18–19 min at 75%–0% B; and 19–24 min at 0% B. The injection volume was set to 2 μL. The mass parameters were as follows: ion spray voltage was 5500 V, ion source temperature was 500 °C, collision gas was set to Medium, ion source gas 1 was 50 psi, ion source gas 2 was 60 psi, curtain gas was 35 psi. Multiple reaction monitoring (MRM) was used to monitor target ribonucleosides in the positive ion mode. The detailed MRM transitions were as follow: A, *m*/*z* 268 → 136; m1A, *m*/*z* 282 → 150; G, *m*/*z* 284 → 152; m1G, *m*/*z* 298 → 166; C, *m*/*z* 244 → 112;m3C, *m*/*z* 258 → 126. The dwell time for each ribonucleoside was 100 ms. The declustering potential and collision energy were 20 and 15 V, respectively. Data acquisition and processing were performed using Analyst (version 1.6, SCIEX).

### qRT-PCR

RNA was reverse transcribed into cDNA with HiScript II 1st Strand cDNA Synthesis Kit (Vazyme) according to manufacturer’s instructions. Quantitative real-time PCR was performed with ChamQ Universal SYBR qPCR Master Mix (Vazyme) on ABI QuantStudio 7 real-time PCR system (Applied Biosystems). Primer sequences are provided in Supplementary Table S4.

### ASO administration into cells

The antisense oligonucleotides (ASOs), targeting U1-5′ snsRNA (5′-GCAGGGGAGATACCATGATCAC-3′), or negative control were synthesized from RiboBio (Guangzhou, China). 10 nM final concentration ASO were transfected using Lipofectamine 2000 (Life Technologies) according to the manufacturer’s instructions. After 72 h, the transfected cells were harvested for total RNA preparation using mirVana miRNA Isolation Kit.

### Immunofluorescence staining

For immunofluorescent staining, the cells were fixed with 4% paraformaldehyde for 15 min at room temperature, and then incubated with 3% BSA-PBS containing 0.25% Triton X-100 (Sigma) for 15 min. Cells were then washed three times with PBS. After being blocked by 3% BSA in PBS for 60 min at room temperature, cells were incubated with Goat anti-Human Albumin Antibody (Bethyl Laboratories, Inc.) for 2 h at room temperature, washed three times with TBST, and then incubated with Cy3-conjugated AffiniPure Donkey Anti-Goat secondary antibody (Jackson) for 60 min at room temperature in dark. Nuclei were stained with DAPI (Sigma). Primary and secondary antibodies were diluted in PBS containing 3% BSA.

### Preparation of probes

The DNA probes were labeled by digoxigenin (DIG) using DIG Oligonucleotide Tailing Kit (2nd Generation, Roche) according to the manufacturer’s instructions. Short tail-labeled probes were generated with 2–3 nucleotides consisting of DIG-dUTP. A mixture of 2 μl of reaction buffer, 2 μl of CoCl_2_-solution, 0.5 μl of DIG-dUTP solution, 0.5 μl of 400 U Terminal transferase, and 100 pmol of oligonucleotides was prepared and briefly centrifuged, followed by incubation at 37 °C for 15 min and cool down on ice. The probes were stored at −20 °C.

### Northern blotting analysis

For Northern blotting, small RNA sample was mixed with Gel loading buffer II (Invitrogen) and incubated at 90 °C for 5 min. Then the samples were incubated on ice for 3 min and loaded into denaturing 15% polyacrylamide gel containing 8 M Urea. The RNAs were transferred onto a positive charged nylon membrane, and UV cross-linked at 150 mJ/cm^2^. Then the membrane was pre-hybridization for 1 h and blotted with DIG-labeled DNA probes against target RNA subsequently, and incubated overnight at 35 °C. The membranes were washed three times with low stringent buffer (2× SSC buffer with 0.1%wt/vol SDS) at room temperature for 10 min each, then rinsed three times with high stringent buffer (0.1× SSC buffer with 0.1%wt/vol SDS) for 10 min each, finally rinsed in 1× DIG washing buffer (Roche) for 10 min. Following the washes, the membranes were incubated with 1× blocking buffer (dilute the 10× blocking solution with 1× Maleic acid buffer, Roche) at room temperature for 1–2 h, after which the DIG antibody (Anti-Digoxigenin-AP Fab fragments, Roche) was added into the blocking buffer at a ratio of 1:10,000 and incubated for additional 2 h at room temperature. The membranes were then washed three times in DIG washing buffer for 15 min each and rinsed in 1× DIG detection buffer (Roche) for 5 min, and then soaked with CSPD ready-to-use reagent (Roche) before imaging using a GE AI680 imaging system. The probe sequences were listed in Supplementary Table S4.

### tRNA aminoacylation analysis

tRNA aminoacylation was determined by acid urea polyacrylamide gel electrophoresis (acid urea PAGE) followed by northern blotting method^49^. Briefly, RNA samples were isolated with Trizol and dissolved in 10 mM sodium acetate solution (pH = 5.2). Then samples were treated with 0.1 M Tris (pH = 9.0) at 37 °C for 45 min, then the treated and control RNA samples were precipitated with 2.5 volume of ethanol and 1/10 volume of 3 M sodium acetate solution (pH 5.2), and resuspended in 10 mM sodium acetate (pH = 5.2). Two micrograms of RNA samples were loaded into 6% acid (pH = 5.2) urea polyacrylamide gel, then the RNAs were separated by electrophoresis using 0.1 M sodium acetate (pH = 5.2) as electrophoresis buffer. After electrophoresis, the RNAs in the gel were transferred onto a positive charged nylon membrane for further northern blotting analysis.

### Pretreatment of small RNA for CPA-seq

Two micrograms of small RNA from each sample was incubated with deacylation buffer (pH = 9.0) at 37 °C for 45 min and followed by ethanol precipitation. The small RNA was recovered in 20 µL nuclease-free water (Ambion). Then the recovered small RNA was treated with TURBO DNA-free kit (Ambion) for DNA contamination removing. The small RNA was purified from the reaction by ethanol precipitation. Then the recovered small RNA was incubated with 1 U Cap-Clip Acid Pyrophosphatase (Cellscript) in 1× Cap-Clip Acid Pyrophosphatase reaction buffer (Cellscript) at 37 °C for 30 min. Then, the reaction was added with 20 U T4 PNK (NEB) in 1× T4 PNK reaction buffer (NEB) and 1 mM ATP (NEB) and incubated at 37 °C for 30 min. The small RNA was purified from the reaction by phenol-chloroform extraction and ethanol precipitation. Then the purified small RNA was treated with 2× molar ratio of AlkB and 4× molar ratio of AlkB (D135S) at 25 °C for 1 h with 300 mM KCl, 2 mM MgCl_2_, 10 µM of (NH4)2Fe(SO4)2·6H_2_O, 300 µM 2-ketoglutarate (2-KG), 2 mM L-ascorbic acid, 50 µg/ml BSA, 50 mM MES buffer (pH 5.0). The reaction was quenched by addition of 5 mM EDTA. After phenol-chloroform extraction and ethanol precipitation, the small RNA was recovered in 3 µL nuclease-free water (Ambion).

### Library preparation for commercially available sRNA library preparation kits

For sequencing of equimolar synthetic small RNAs (Universal Reference, MACS), 5 fmol of equimolar synthetic small RNAs were used for library preparation. For sequencing of small RNAs from HEK293T cells, 50 ng of small RNA were used for library preparation of each sample. NEBNext Small RNA kit (NEB), TruSeq Small RNA Library Prep Kit and QIAseq miRNA Library Kit were used for preparation small RNA libraries according to manufacturer’s recommendations.

### Preparation of adenylated 3′ DNA adapter

The 3′ DNA adapter was adenylated according to the introduction of the 5′ DNA Adenylation Kit (NEB). 10 pmol synthetic 5′-phosphate 3′ DNA adapter with 2 µL 10× 5′ DNA adenylation reaction Buffer, 2 µL 1 mM ATP, 2 µL Mth RNA Ligase in 20 µL reaction solution were incubated at 65 °C for 3 h. The adenylated 3′ DNA adapter was stored at −80 °C until use.

### Library preparation for CPA-seq

For 3′ adapter ligation, 5 fmol of equimolar synthetic small RNAs or 50 ng of pretreated small RNA from each sample of HEK293T cells and mouse tissues were mixed with 10 pmol of adenylated 3′ DNA adapter and nuclease-free water to a volume of 4 µL, and preincubated at 70 °C for 2 min. The reaction was transferred to ice and incubated for 5 min. The reaction was initiated by adding 200 U T4 RNA ligase 2 truncated KQ (NEB), 40 U murine RNase inhibitor (NEB), 3 µL 50% PEG 8000 (NEB), and 1 µL of 10× T4 RNA ligase reaction buffer (NEB) to a final volume of 10 µL. The reaction was incubated at 25 °C for 2 h. To remove 3′ adapters, 50 U of 5′ Deadenylase (NEB) was added to the reaction, and the reaction was incubated at 30 °C for 1 h, followed by adding 1 µL of RecJf (NEB) and incubating at 37 °C for 1 h. Then the reaction was incubated at 70 °C for 20 min to inactivate enzymes used in 3′ adapter ligation. Before 5′ adapter ligation, the 5′ RNA adapters were denatured at 70 °C for 2 min and immediately transferred to ice. To initiate 5′ adapter ligation, the reaction resulting from 3′ adapter ligation was mixed with 1 µL of 25 μM denatured 5′ RNA adapter, 1 µL of 10 mM ATP (NEB), 1 µL of T4 RNA Ligase 1 (ssRNA Ligase, 30 units/µL, NEB) to a volume of 15 µL, and incubated at 37 °C for 2 h. Next, the reaction was mixed with 1 µL of 10 µM reverse transcription (RT) primers, heated at 75 °C for 5 min, and then incubated at 37 °C for 15 min, followed by incubation at 25 °C for 15 min to hybridize the RT primers. To perform reverse transcription, the reaction resulting from hybridization of RT primers was mixed with 1.7 µL of 5 M NaCl, 1 µL of 25 mM dNTPs (an equimolar mix of 25 mM dATP, dCTP, dGTP, and dTTP), 1 µL of 100 mM Dithiothreitol, 0.7 µL of murine RNase inhibitor, and 1 µL of 200 units/µL TGIRT-III enzyme (Index) to a final volume of 21.4 µL, and incubated at 57 °C for 2 h. The cDNAs resulting from reverse transcription were mixed with 2× TBE-Urea loading buffer and incubated at 90 °C for 5 min. Then the samples were incubated on ice for 3 min, and loaded into denaturing 15% polyacrylamide gel containing 8 M Urea for electrophoresis. The bands corresponding to libraries of RNA between 15 and 50 nt were sized selected, and purified by ethanol precipitation. The purified cDNAs were recovered in 23 µL nuclease-free water.

To perform PCR amplification, 23 µL of purified cDNAs were mixed with 25 µL of NEBNext Ultra II Q5 Master Mix, 1 µL of 10 mM SR Primer for Illumina, 1 µL of 10 mM Index Primer to a final volume of 50 µL. The PCR reaction was performed for 15 cycles of 98 °C for 10 s, 61 °C for 30 s, and 72 °C for 15 s. The PCR products were electrophoresed in a 6% polyacrylamide gel. The band corresponding to PCR products between 140 and 200 bp was size selected and purified by ethanol precipitation. Then the PCR products were sequenced using Illumina HiSeq X10 paired-end 2 × 150 bp sequencing. The sequences of adapters and primers were listed in Supplementary Table S4.

### Preprocessing and read counting of sRNA-seq data

Trim_galore (0.6.4) was used to remove the adapter sequences and sequencing reads with QC < 30. The sequences corresponding to small RNAs between 15 and 40 nt were used for subsequent analyses. For sequencing reads from CPA-seq, umitools^50^ was used to remove PCR duplicates and generate unique UMI reads. To accelerate the sequence alignment, identical sequences were collapsed together for read counting and subsequent mapping.

### sRNA annotation

Bowtie (version 1.0.0, --norc -k 1) was used for the reads mapping. We allowed zero mismatch for miRNA mapping to avoid the misannotation of some tsRNAs that share similar sequences with miRNAs. For other reads mapping, one mismatch was allowed. The sequencing reads were mapped to the miRNA, rRNA, cytosolic tRNA, tRNA precursor, piRNA cluster, ncRNA, and genome in order. For miRNA mapping, reads between 16 and 28 nt were selected and mapped to the miRBase. To generate reference sequences for mature cytosolic tRNAs, we added a CCA sequence to the 3′-ends of all tRNA reference sequences and a G to the 5′-ends of histidine tRNAs. To generate reference sequences for tRNA precursors, we extracted sequences from 100 bp upstream to 100 bp downstream of the tRNAs in genome. To annotate the types of tsRNAs, we used MINTmap^51^. For piRNA mapping, reads between 24 and 32 nt were selected and mapped to the piRNA clusters. The reference genome sequences (human: hg38, mouse: mm10) were downloaded from the UCSC (https://genome.ucsc.edu/). The miRNA reference sequences were from miRbase v22.0 (http://www.mirbase.org/). The tRNA reference sequences were from GtRNAdb (http://gtrnadb.ucsc.edu/). The 5S rRNA reference sequences were from 5S rRNA database (http://combio.pl/rrna/). The 5.8S, 18S, 28S rRNAs, and 45S rRNA reference sequences were from SILVA (https://www.arb-silva.de/) and NCBI (https://www.ncbi.nlm.nih.gov/). The snRNA, snoRNA, lncRNA, and other ncRNA reference sequences were from Ensembl (https://asia.ensembl.org/index.html). The piRNA cluster reference sequences were from piRNA Cluster DataBase (https://www.smallrnagroup.uni-mainz.de/piCdb/).

### Analysis of sRNA expression

To compare the expressions of sRNAs, we used the RPM (reads per million mapped reads). We then find the highest expression sequences in residual sequences as the next parental sequence until we find out all sequences. Considering the efficiency during the human MSC^LT^ to hepatocyte-like cells, we select small RNAs which are highly detected in primary human hepatocyte compared to human MSC^LT^ (*t*-test, *P* value < 0.05 and fold change > 2) to describe the sRNomes in hepatic reprogramming. We compute the Euclidean Distance for log_2_ transformed unique reads to get the hierarchically-clustered heatmap. We plot the *t*-distributed stochastic neighbor embedding (*t*-SNE) of different tissues by an R package (Rtsne) with initial_dims = 100 and maxiter = 1000. Only the unique reads with RPM > 20 in at least one tissue are used for clustering, *t*-SNE plot, and correlation analysis. R (3.6.2) and Python (3.6.7) were used for statistical analysis.

### Tissue specificity index

Tissue specificity index (TSI) was used to evaluate the expression variability of each sRNA across different mouse tissues as previously described^42^. The formula used for computing the TSI is:

$${\mathrm{TSI}}_j = \frac{{N - \frac{{\mathop {\sum }\nolimits_{i = 1}^N x_{j,i}}}{{\mathop {{\max }}\limits_i x_{j,i}}}}}{{N - 1}}$$

Where *N* is the total number of tissues measured and *x*_*j,i*_ is the expression intensity of sRNA *j* in tissue *i*.

## Supplementary information

Table S4

Table S5

Fig S1

Fig S2

Fig S3

Fig S4

Fig S5

Fig S6

Fig S7

Table S1

Table S2

Table S3

## Data Availability

Raw sequencing data were stored in Sequence Read Archive (SRA), under accession number PRJNA633608.
