# Supplementary material for: CPA-seq reveals small ncRNAs with methylated nucleosides and diverse termini
Source: Cell Discov. 2021 Apr 19;7:25. doi: 10.1038/s41421-021-00265-2 (PMC8053708; doi:10.1038/s41421-021-00265-2)
Supplement: Supplementary file 1 — Table S4 [file 41421_2021_265_MOESM1_ESM.pdf]

Supplementary Table S4. List of oligo sequences used in library preparation and Supplementary Fig. 1a-c, and probes used for Northern blotting.

Oligos for CPA-seq	Sequence
5’ adapter	5'-GUUCAGAGUUCUACAGUCCGACGAUC(N:25:25:25:25:25:25)(N)(N)(N) (N)(N)(N)-3'
3’ adapter	5'-P-(N:25:25:25:25:25:25:25:25:25)(N)(N)(N)(N)(N)(N)(N)(N)(N) AGATCGGAAGAGCACACGTC-3ddC-3'
RT primer	5'-A-s-G-s-A-s-C-s-G-s-TGTGCTCTTCCGATCT-3'
PCR primer	5'-AATGATACGGCGACCACCGAGATCTACACGTTCAGAGTTCTACAGTCCG-s-A-3'
PCR Index primer	5´-CAAGCAGAAGACGGCATACGAGATXXXXXXGTGACTGGAGTTCAGACGTGTGCTCTTCCGATC-s-T-3´
**Adapters for Fig.1**	**Sequence**
5’ adapter	5'-GUUCAGAGUUCUACAGUCCGACGAUC-3'
3’ adapter	5'-P-(N:25:25:25:25:25:25:25:25:25)(N)(N)(N)(N)(N)(N)(N)(N)(N)AGATCGGAAGAGCACACGTC-3ddC-3'
**RNAs for Fig.1 and Supplementary Fig.S1**	**Sequence**
5’-OH-RNA	5'-CGGUACUGCAGCUGACCUCGGCUUGUG-3'
3’-P-RNA	5'-CGGUACUGCAGCUGACCUCGGCUUGUG-P-3'
5'N7-Methylguanosine-triphosphate Cap RNA	5'-M7GpppAUACUUACCUGGCAGGGGAGAUACCAUGAU-3'
**Probes for Northern Blotting**	**Sequence**
iMet-CAT-3'end	TGGTAGCAGAGGATGGTTT
iMet-CAT-5'end	GCTTCCGCTGCGCGACTCTGCT
Gly-CCC-3'end	GAACCCGGGTCGCAAGAAT
Gly-CCC-5'end	CATGATACCACTACACCAGCGGCGC
Arg-CCT(19-42nt)	TGGCTTAGGAGGCCAGTGCCTTAT
Leu-CAG-3'end	GGTGTCAGGAGTGGGATTCG

Leu-CAG-5'end	GAACGCAGTGCCTTAGACCGCTCGGCCATCCTGAC
Val-CAC-5'end	GAGGCGAACGTGATAACCACTACACTACGGAAAC
Val-CAC-3'end	TTTCGAACCGGGGACCTTTCGCGT
Pro-TGG-5'end	CATACCCCTAGACCAACGAGCC
RNU1-5'end	TGGTATCTCCCCTGCCAGGTAAGTAT
Mouse-Trp-CCA	GGTGACCCCGACGTGATTCGAA
Ser-GCT-5‘end	AACCACTCGGCCACCTCGTC
Ser-GCT-3’-end	ACGAGGATGGGATTCGAACCCAC
Glu-CTC_5‘end	GAGCGCCGAATCCTAACCACTAGACCACCAGGGA
Glu-CTC-3’end	GAACCCGGGCCGCGGCGGT
Gln-TTG-5‘end	GCTAACCATTACACCATGGGACC
Cys-GCA-3’end	GGAGGGGGCACCCGGATAT
Cys-GCA-5‘end	TGCTCTGCCCCTGAGCTATACCC
MT-TV	TTTGTGTTAAGCTACACTCTG
**qPCR Primers**	**Sequence**
ASGPR1-F	ATGACCAAGGAGTATCAAGACCT
ASGPR1-R	TGAAGTTGCTGAACGTCTCTCT
Transferrin-F	TGTCTACATAGCGGGCAAGT
Transferrin-R	GTTCCAGCCAGCGGTTCT
ALB-F	GCCTTTGCTCAGTATCTT
ALB-R	AGGTTTGGGTTGTCATCT

s = phosphorothioate bond.

XXXXXX = index sequence.
