# Supplementary material for: CPA-seq reveals small ncRNAs with methylated nucleosides and diverse termini
Source: Cell Discov. 2021 Apr 19;7:25. doi: 10.1038/s41421-021-00265-2 (PMC8053708; doi:10.1038/s41421-021-00265-2)
Supplement: Supplementary file 2 — Table S5 [file 41421_2021_265_MOESM2_ESM.pdf]

Supplementary Table S5. List of RNA integrity number (RIN) values of samples.

Tissue	RIN value (sample #1)	RIN value (sample #2)
Heart	9.4	9.1
Liver	9.0	9.1
Lung	8.6	8.8
Stomach	9.1	9.2
Kidney	9.2	9.3
Testis	9.3	9.4
Whole brain	8.8	8.8
Ovary	10.0	10.0
Muscle	9.2	9.1
**MSC^{LT}+FHH**	**RIN value (sample #1)**	**RIN value (sample #2)**
Day 0	10.0	10.0
Day 3	9.9	9.9
Day 6	9.8	9.8
Day 14	9.6	9.4
PHH	9.6	9.3
