# Supplementary material for: CPA-seq reveals small ncRNAs with methylated nucleosides and diverse termini
Source: Cell Discov. 2021 Apr 19;7:25. doi: 10.1038/s41421-021-00265-2 (PMC8053708; doi:10.1038/s41421-021-00265-2)
Supplement: Supplementary file 3 — Fig S1 [file 41421_2021_265_MOESM3_ESM.pdf]

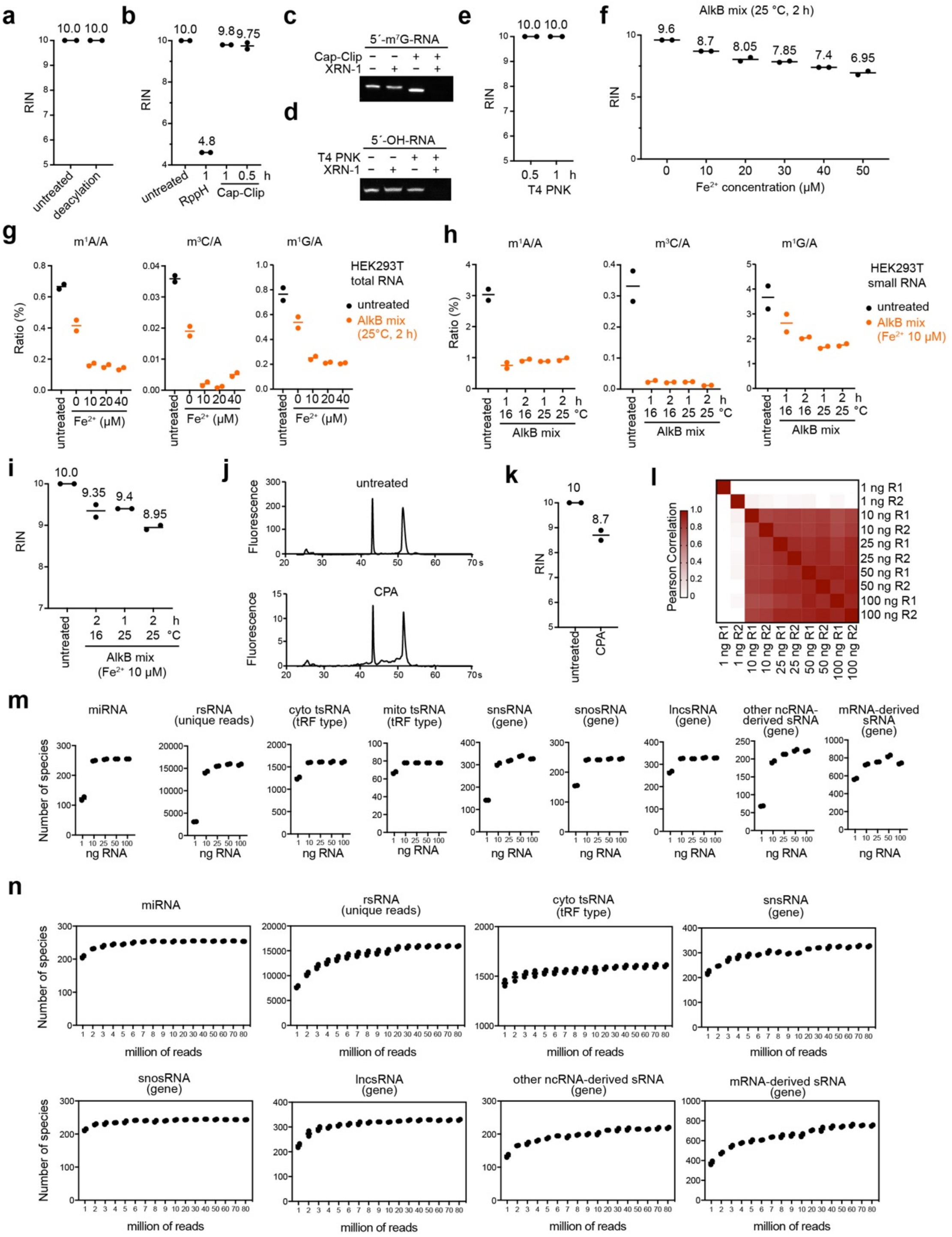
a
RIN
untreated
deacylation
b
RppH
Cap-Clip
c
5´-m7G-RNA
Cap-Clip
XRN-1
d
5´-OH-RNA
T4 PNK
XRN-1
e
T4 PNK
f
AlkB mix (25 °C, 2 h)
Fe2+ concentration (μM)
g
m1A/A
m3C/A
m1G/A
Ratio (%)
Fe2+ (μM)
HEK293T total RNA
untreated
AlkB mix (25°C, 2 h)
h
HEK293T small RNA
AlkB mix (Fe2+ 10 μM)
AlkB mix
i
AlkB mix (Fe2+ 10 μM)
j
untreated
CPA
Fluorescence
k
l
Pearson Correlation
m
miRNA
rsRNA (unique reads)
cyto tsRNA (tRF type)
mito tsRNA (tRF type)
snsRNA (gene)
snosRNA (gene)
lncsRNA (gene)
other ncRNA-derived sRNA (gene)
mRNA-derived sRNA (gene)
Number of species
ng RNA
n
million of reads

Supplementary Fig. S1. Optimization of CPA-seq.

a. The RNA integrity number (RIN) of total RNAs from HEK293T cells with/w.o. treatment of deacylation buffer (n = 2). The RIN values are generated by bioanalyzer. **b.** The RIN values of total RNAs from HEK293T cells with indicated treatments. Decapping enzyme Cap-Clip is better than RppH in preserving RNA integrity (n = 2). **c.** Treatment of Cap-Clip enables synthetic 5´-m7G-RNA to be digested by XRN-1, a 5´→3´ exoribonuclease that requires 5´-P. **d.** Treatment of T4 PNK enables synthetic 5´-OH-RNA to be digested by XRN-1. **e.** The RIN values of total RNAs from HEK293T cells treated with T4 PNK for indicated time (n = 2). **f.** The RIN values of total RNAs from HEK293T cells treated with AlkB mix in the buffer containing indicated concentration of Fe^{2+} (n = 2). **g.** LC-MS/MS analysis showing the demethylation efficiency of AlkB mix in the buffer containing indicated concentration of Fe^{2+} (n = 2). **h.** LC-MS/MS analysis showing the demethylation efficiency of AlkB mix under indicated temperature for indicated incubation time (n = 2). **i.** The RIN values of total RNAs from HEK293T cells treated with AlkB mix under indicated temperature for indicated incubation time (n = 2). **j.** Representative bioanalyzer electropherograms of total RNAs from HEK293T cells with/w.o. CPA treatment. **k.** The RIN values of total RNAs from HEK293T cells with/w.o. CPA treatment (n = 2). **l.** Pearson correlation heat map of the sRNA species generated from 1 to 100 ng of small RNA of HEK293T cells using CPA-seq (n = 2). **m.** Species numbers of different sRNA types detected from 1 to 100 ng of small RNA of HEK293T cells using CPA-seq (n = 2). **n.** Species numbers of different sRNA types generated from 1 to 80 million reads of CPA-seq of small RNA from HEK293T cells (n = 2).
