# Supplementary material for: CPA-seq reveals small ncRNAs with methylated nucleosides and diverse termini
Source: Cell Discov. 2021 Apr 19;7:25. doi: 10.1038/s41421-021-00265-2 (PMC8053708; doi:10.1038/s41421-021-00265-2)
Supplement: Supplementary file 4 — Fig S2 [file 41421_2021_265_MOESM4_ESM.pdf]

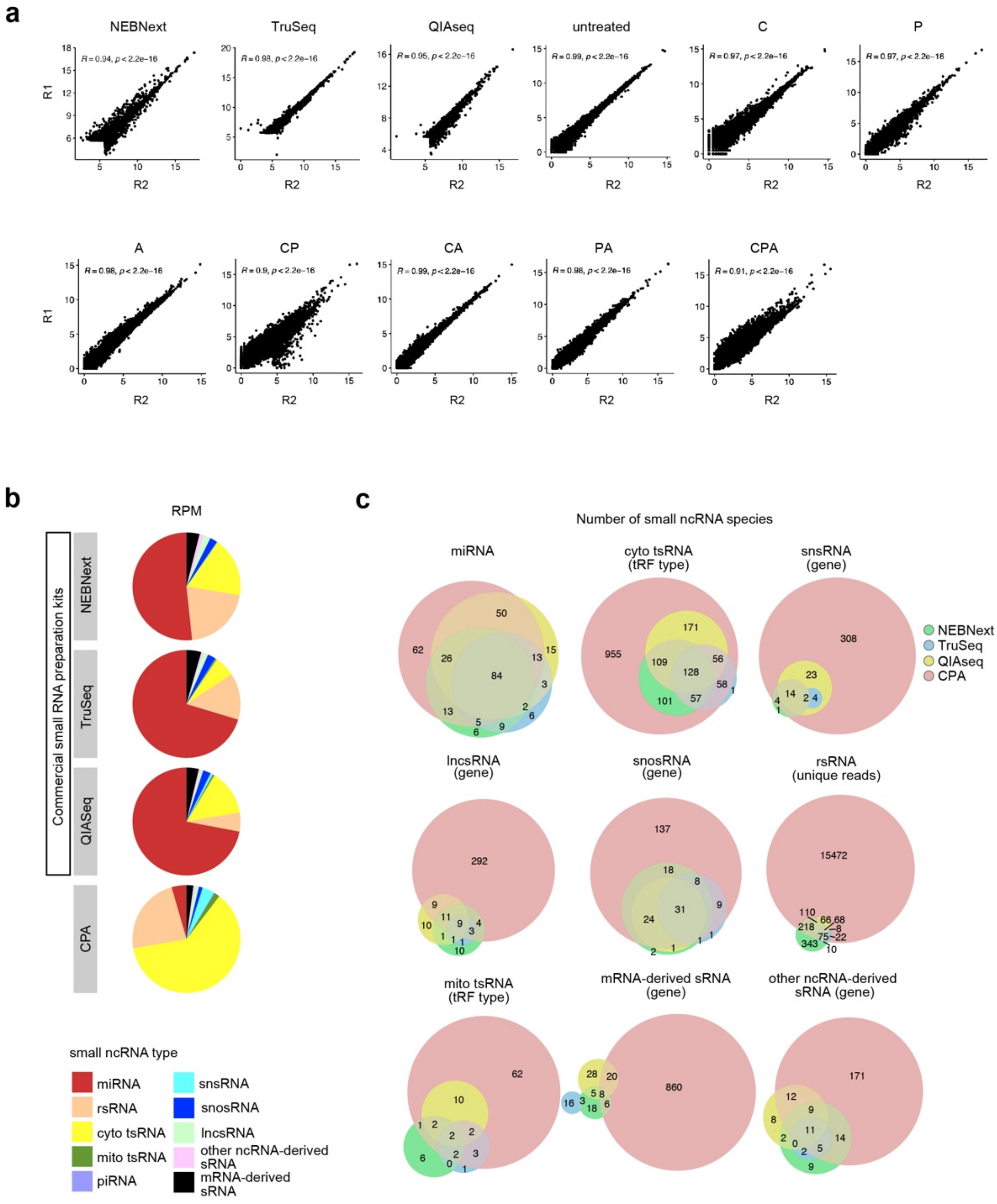

Supplementary Fig. S2. Comparison of different sRNA-seq library preparation methods.

a. Scatter plots with Pearson correlation assessing the reproducibility between technique replicates of indicated sRNA-seq methods (RPM > 50). **b.** Distribution of different types of sRNAs of HEK293T cells revealed by different sRNA-seq methods (n = 2). **c.** Venn diagrams show the species numbers of sRNAs (Normalized RPM > 50, RPM values are normalized to total miRNA RPM) revealed by different sRNA-seq methods (n = 2).
