# Supplementary material for: CPA-seq reveals small ncRNAs with methylated nucleosides and diverse termini
Source: Cell Discov. 2021 Apr 19;7:25. doi: 10.1038/s41421-021-00265-2 (PMC8053708; doi:10.1038/s41421-021-00265-2)
Supplement: Supplementary file 5 — Fig S3 [file 41421_2021_265_MOESM5_ESM.pdf]

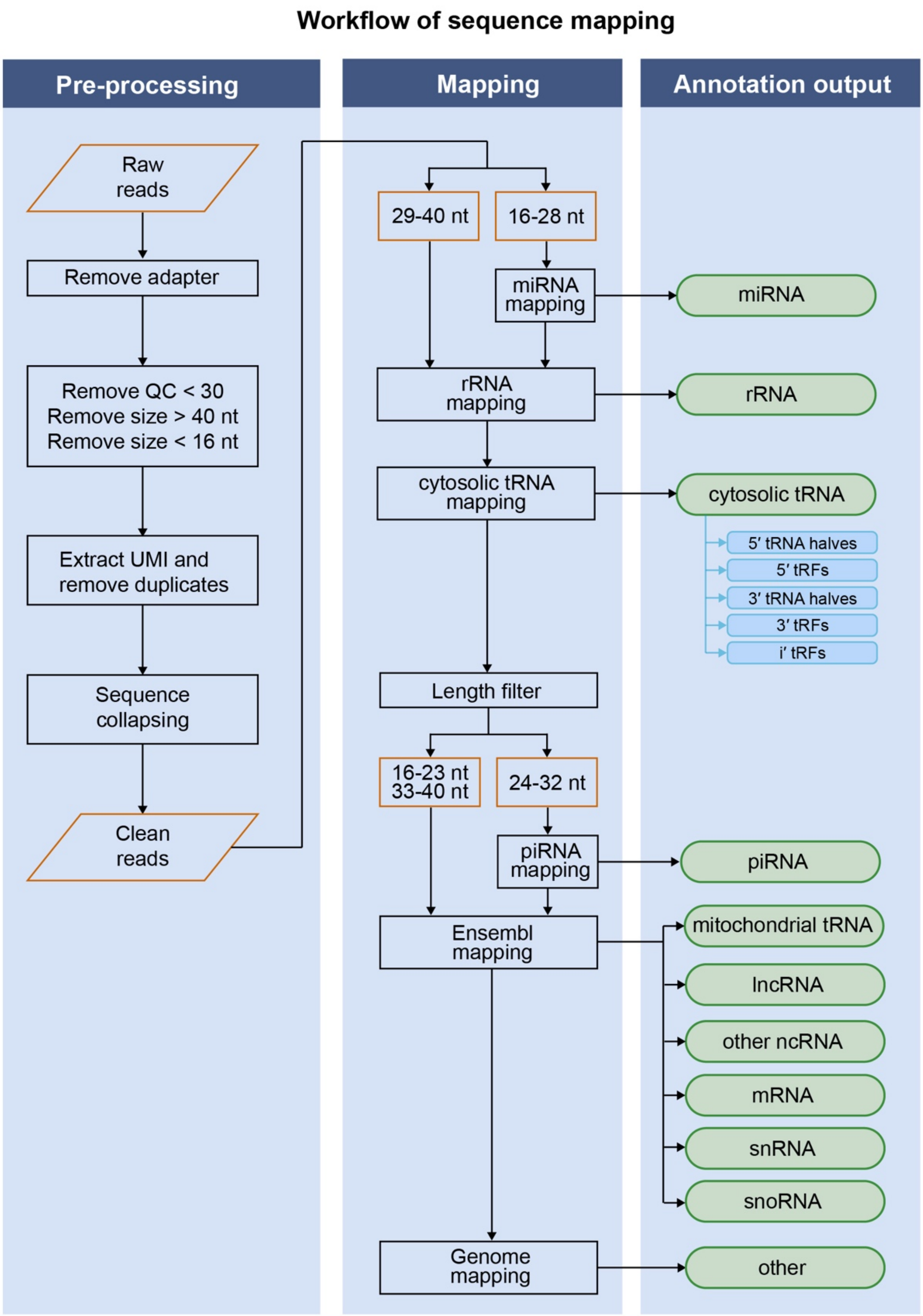


**Supplementary Fig. S3. Workflow for the mapping of sequencing reads.**

The sequencing reads were trimmed and filtered, followed by sequential alignment with each library for annotation.
