# Supplementary material for: CPA-seq reveals small ncRNAs with methylated nucleosides and diverse termini
Source: Cell Discov. 2021 Apr 19;7:25. doi: 10.1038/s41421-021-00265-2 (PMC8053708; doi:10.1038/s41421-021-00265-2)
Supplement: Supplementary file 6 — Fig S4 [file 41421_2021_265_MOESM6_ESM.pdf]

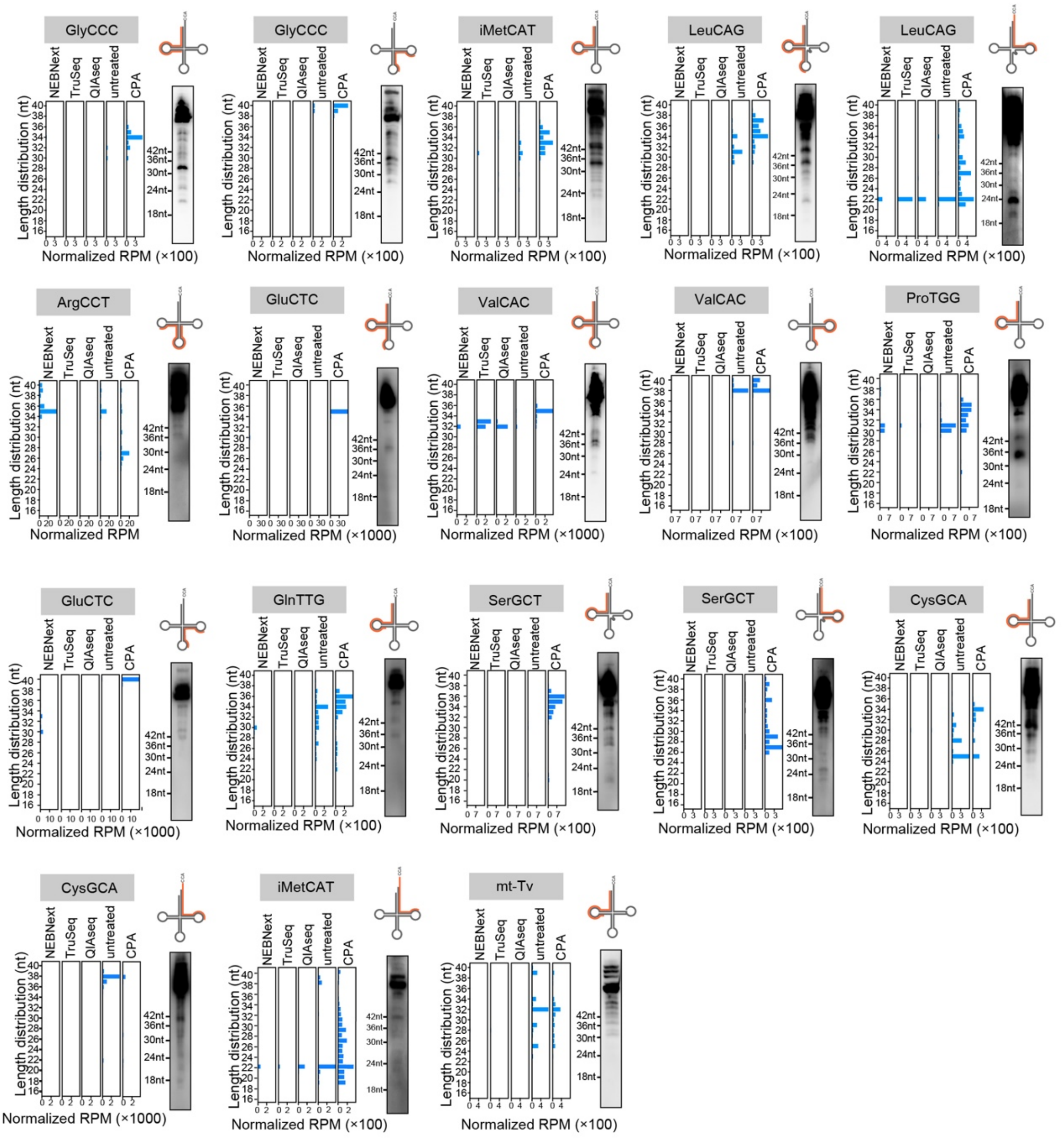

Supplementary Fig. S4. Verification of sRNAs detected by CPA-seq.

Northern blotting validation of tsRNAs. The length distributions of RPM of sRNA reads containing the probe (orange) sequence (mismatch ≤ 2 nt, *n* = 2) are shown on the left.
