# Supplementary material for: CPA-seq reveals small ncRNAs with methylated nucleosides and diverse termini
Source: Cell Discov. 2021 Apr 19;7:25. doi: 10.1038/s41421-021-00265-2 (PMC8053708; doi:10.1038/s41421-021-00265-2)
Supplement: Supplementary file 7 — Fig S5 [file 41421_2021_265_MOESM7_ESM.pdf]

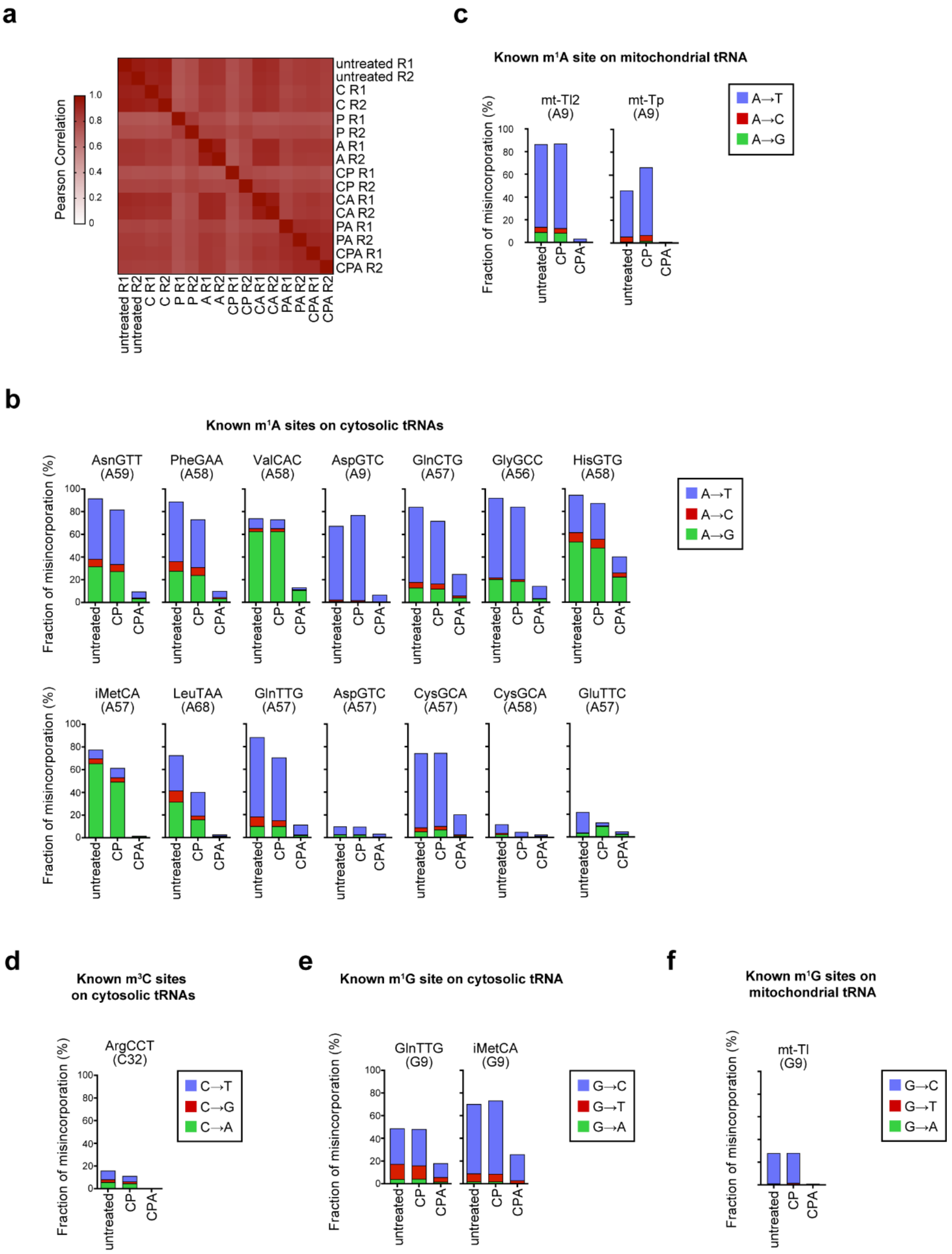
a
Pearson Correlation
1.0
0.8
0.6
0.4
0.2
0
untreated R1
untreated R2
C R1
C R2
P R1
P R2
A R1
A R2
CP R1
CP R2
CA R1
CA R2
PA R1
PA R2
CPA R1
CPA R2
c
Known m1A site on mitochondrial tRNA
mt-Tl2 (A9)
mt-Tp (A9)
A→T
A→C
A→G
Fraction of misincorporation (%)
untreated
CP
CPA
b
Known m1A sites on cytosolic tRNAs
AsnGTT (A59)
PheGAA (A58)
ValCAC (A58)
AspGTC (A9)
GlnCTG (A57)
GlyGCC (A56)
HisGTG (A58)
iMetCA (A57)
LeuTAA (A68)
GlnTTG (A57)
AspGTC (A57)
CysGCA (A57)
CysGCA (A58)
GluTTC (A57)
A→T
A→C
A→G
Fraction of misincorporation (%)
untreated
CP
CPA
d
Known m3C sites on cytosolic tRNAs
ArgCCT (C32)
C→T
C→G
C→A
Fraction of misincorporation (%)
untreated
CP
CPA
e
Known m1G site on cytosolic tRNA
GlnTTG (G9)
iMetCA (G9)
G→C
G→T
G→A
Fraction of misincorporation (%)
untreated
CP
CPA
f
Known m1G sites on mitochondrial tRNA
mt-Tl (G9)
G→C
G→T
G→A
Fraction of misincorporation (%)
untreated
CP
CPA

Supplementary Fig. S5. Methylomes of sRNAs in HEK293T cells.

a. Pearson correlation heat map of miRNAs generated from sRNA with indicated treatments. MiRNAs revealed by different combinations of Cap-Clip, T4 PNK and AlkB mix treatment show high correlation. For the following analysis of CPA-seq of HEK293T cells, RPM values were normalized to total miRNA RPM. **b-f.** The mismatch frequencies at known m^1A sites of cytosolic tRNAs (**b**) and mitochondrial tRNAs (**c**), known m^3C sites of cytosolic tRNAs (**d**), known m^1G sites of cytosolic tRNAs (**e**) and mitochondrial tRNAs (**f**) (n = 2).
