# Supplementary material for: CPA-seq reveals small ncRNAs with methylated nucleosides and diverse termini
Source: Cell Discov. 2021 Apr 19;7:25. doi: 10.1038/s41421-021-00265-2 (PMC8053708; doi:10.1038/s41421-021-00265-2)
Supplement: Supplementary file 8 — Fig S6 [file 41421_2021_265_MOESM8_ESM.pdf]

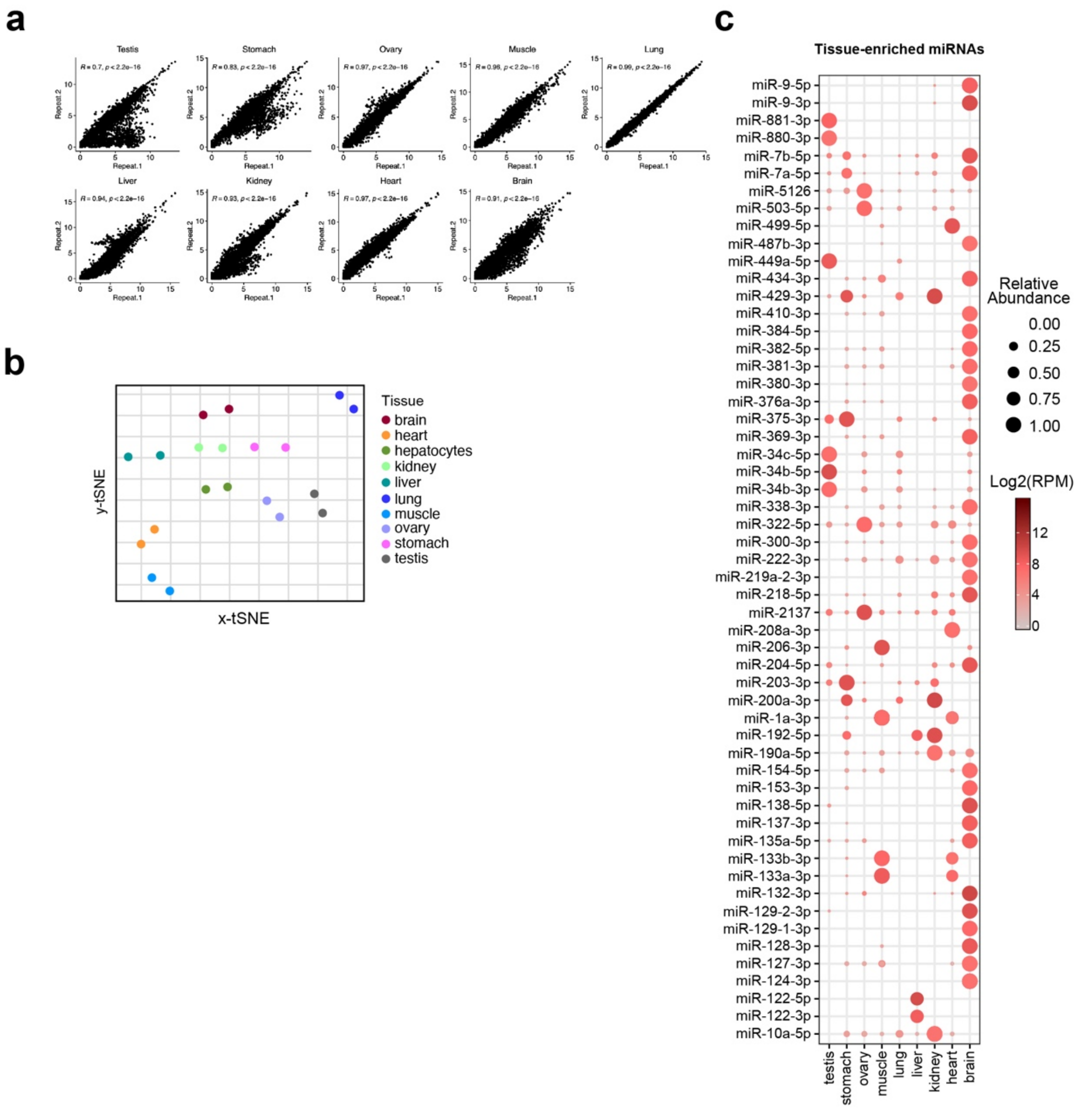

Supplementary Fig. S6. Analysis of sRNAs in different mouse tissues.

a. Scatter plots with Pearson correlations assessing the reproducibility between biological replicates of each tissue. **b.** t-SNE projection plots showing that the sRNAs were clustered according to their tissue type. **c.** Dot plot showing expression patterns of tissue-specific miRNAs (*TSI* > 0.9, RPM > 100 in at least one tissue).
