# Supplementary material for: CPA-seq reveals small ncRNAs with methylated nucleosides and diverse termini
Source: Cell Discov. 2021 Apr 19;7:25. doi: 10.1038/s41421-021-00265-2 (PMC8053708; doi:10.1038/s41421-021-00265-2)
Supplement: Supplementary file 9 — Fig S7 [file 41421_2021_265_MOESM9_ESM.pdf]

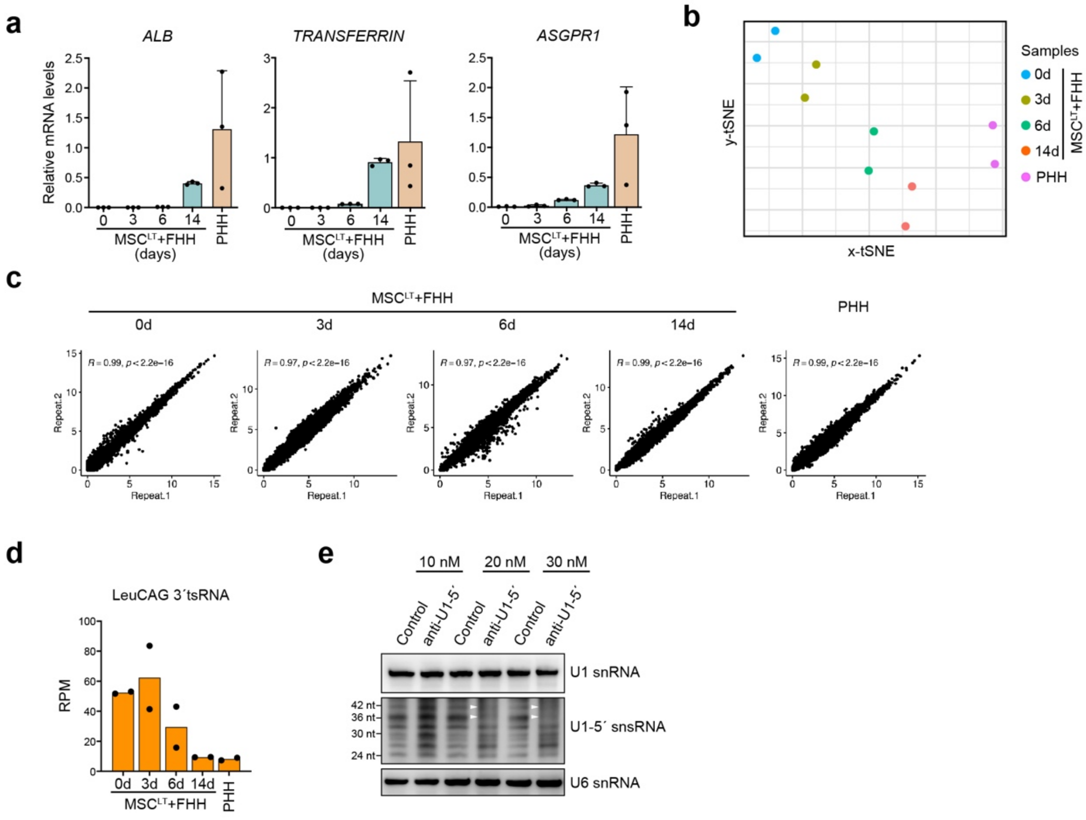

Supplementary Fig. S7. sRNomes of MSC^{LT} undergoing hepatic reprogramming.

a. Quantitative PCR showing the induction of hepatic genes in MSC^{LT} undergoing hepatic reprogramming. **b.** t-SNE projection plots showing the reprogramming of sRNA patterns of MSC^{LT} cells transfected with FHH. **c.** Scatter plots with Pearson correlations assessing the reproducibility between biological replicates of each sample. **d.** Differential expression levels of LeuCAG 3´tsRNA in PHH and MSC^{LT} overexpressing FHH for indicated days. The expression levels of LeuCAG 3´tsRNA gradually decreased in MSC^{LT} cells infected with FHH (n = 2). **e.** Northern blotting showing the knock down of U1-5´snsRNAs by an U1-5´ ASO in HEK293T.
