# Supplementary material for: CPA-seq reveals small ncRNAs with methylated nucleosides and diverse termini
Source: Cell Discov. 2021 Apr 19;7:25. doi: 10.1038/s41421-021-00265-2 (PMC8053708; doi:10.1038/s41421-021-00265-2)
Supplement: Supplementary file 10 — Table S1 [file 41421_2021_265_MOESM10_ESM.pdf]

Supplementary Table S1. Read numbers of different types of small ncRNAs in HEK293T cells.

Sample	miRNA	rsRNA	Cyto tsRNA	Mito tsRNA	piRNA	snsRNA	snosRNA	lncsRNA	Other ncRNA-derived sRNA	mRNA-derived sRNA	other	Total
NEB #1	5989109	2996808	3291515	42028	20817	43866	600441	457587	3252205	362842	797912	17855130
NEB #2	5946039	4299365	3181801	39107	20300	38888	500915	515448	3292896	344378	736962	18916099
Truseq #1	7813467	2026781	1105863	61072	20241	17380	402973	305639	3040746	291160	503878	15589200
Truseq #2	17905129	4882381	2364243	141895	46372	43922	970714	1232507	5839337	653835	1174659	35254994
Qiagen #1	2799300	401813	1033691	46980	18939	63525	218265	132117	1356497	196135	489962	6757224
Qiagen #2	2395619	359232	802673	33154	17717	55025	173976	139704	1173314	174720	423261	5748395
untreat #1	357068	490577	768898	86385	10556	443866	196476	151606	427664	236417	598765	3768278
untreat #2	268903	434903	617864	70535	8846	375579	165092	124803	368646	199493	517851	3152515
C #1	178380	314995	445343	47308	26471	269377	117945	125917	248331	165488	366514	2306069
C #2	209321	327336	532185	53410	28334	280227	132082	117104	298923	147175	369305	2495402
P #1	99962	922813	2109106	55245	5172	170819	87437	77016	175410	135992	278988	4117960
P #2	137275	1194895	2669171	64591	6438	211213	103071	89949	242300	127911	287582	5134396
A #1	256673	366192	2154804	99653	6070	200383	104137	96483	314338	130607	318441	4047781
A #2	226083	331151	2074978	103329	5554	189072	105631	81494	274801	117001	300418	3809512
CP #1	73843	851760	1126538	45491	11640	193473	84466	114541	193305	133121	259929	3088107
CP #2	110323	1205626	2882295	62503	13211	224675	92234	102850	209756	131664	277653	5312790
CA #1	299985	718097	3342461	126186	35947	382118	153517	142355	342822	168980	423861	6136329
CA #2	259507	606219	2837856	115952	29280	332608	138196	126779	300837	156465	390611	5294310
PA #1	136392	1200870	3412909	112199	6522	177389	86186	91008	244089	122021	257187	5846772
PA #2	164018	1617696	4081561	124341	7909	243641	89855	90104	242911	140196	293828	7096060
CPA #1	182617	1781178	3129325	119542	16212	256862	90815	123189	253655	177460	322094	6452949
CPA #2	146880	1669185	5269431	144249	14722	271979	103159	102979	242203	147896	319973	8432656
