# Supplementary material for: CPA-seq reveals small ncRNAs with methylated nucleosides and diverse termini
Source: Cell Discov. 2021 Apr 19;7:25. doi: 10.1038/s41421-021-00265-2 (PMC8053708; doi:10.1038/s41421-021-00265-2)
Supplement: Supplementary file 11 — Table S2 [file 41421_2021_265_MOESM11_ESM.pdf]

Supplementary Table S2. Read numbers of different types of small ncRNAs in different mouse tissues.

Sample	miRNA	rsRNA	Cyto tsRNA	Mito tsRNA	piRNA	snsRNA	snosRNA	lncsRNA	Other ncRNA-derived sRNA	mRNA-derived sRNA	other	Total
**testis_1**	167930	4056674	2453253	61217	2841623	254374	42832	765031	171895	657777	541840	12014446
**testis_2**	175330	1800275	1825867	41446	3025529	232191	29415	743440	110612	348458	448388	8780951
**stomach_1**	198829	2627694	2479943	124090	3802	76333	31770	77998	172465	320056	277442	6390422
**stomach_2**	211967	885820	2332516	126710	5766	69686	30986	68188	170569	232647	287143	4421998
**ovary_1**	297485	3948350	2563954	99115	6405	304982	145905	141792	204037	199226	274993	8186244
**ovary_2**	224069	2716465	1821584	85009	5631	273269	116529	113978	147685	141817	260139	5906175
**muscle_1**	500243	1164862	4359285	234589	9136	76315	35540	125320	462265	675567	515763	8158885
**muscle_2**	660600	1019642	4037451	290911	8060	77957	36302	105242	612063	450418	418292	7716938
**lung_1**	478439	2664545	6872025	103851	17360	252421	78983	260601	357035	1221117	767640	13074017
**lung_2**	454455	2517672	5850862	89010	15657	220303	72280	236972	326446	1183009	728515	11695181
**liver_1**	119731	627641	2767213	208653	1170	78024	38092	65982	138274	147242	323424	4515446
**liver_2**	118484	1216174	7384631	388168	1343	119334	54360	85233	155688	143696	282778	9949889
**kidney_1**	404083	682299	2076238	393646	11707	113893	40421	136663	369041	435241	410388	5073620
**kidney_2**	481295	781546	3017365	566140	6854	183816	48907	116834	495439	430929	492666	6621791
**heart_1**	592868	581289	2546606	908796	1346	91795	34483	102289	728825	185558	513047	6286902
**heart_2**	383685	551752	2313330	784332	1517	81165	27278	79387	552778	157551	430174	5362949
**brain_1**	435554	799254	2789313	552795	11546	326248	75441	127730	417528	730065	331844	6597318
**brain_2**	545189	390435	2118263	216158	13108	159913	31407	112047	377955	764669	306700	5035844
