# Supplementary material for: CPA-seq reveals small ncRNAs with methylated nucleosides and diverse termini
Source: Cell Discov. 2021 Apr 19;7:25. doi: 10.1038/s41421-021-00265-2 (PMC8053708; doi:10.1038/s41421-021-00265-2)
Supplement: Supplementary file 12 — Table S3 [file 41421_2021_265_MOESM12_ESM.pdf]

Supplementary Table S3. List of tissue-enriched small ncRNA sequences revealed by CPA-seq.

ID	sequence	symbol_name	TSI	small ncRNA type
ID31	GTTAATGTAGCTTAAT	mt-Tf	1	mito tsRNA
ID36	CATAGTGTAGCTTAATA	mt-Tv	1	mito tsRNA
ID137	ACTTTTATAGGATAAT	mt-Tl2	1	mito tsRNA
ID846	AGAAATATGTCTGATAAAAGAATTACTTTGATAGA	mt-Ti	1	mito tsRNA
ID861	AGAAATATGTCTGATAAAAGAATTACTTTGATAGAG	mt-Ti	1	mito tsRNA
ID869	AGAAATATGTCTGATAAAAGAATTACTTTGATAG	mt-Ti	1	mito tsRNA
ID1213	CTTAAAACCTTGTTCCCAGAGGTTCAAATCCTCTCCA	mt-Tl1	1	mito tsRNA
ID1219	TTAAAACCTTGTTCCCAGAGGTTCAAATCCTCTCCA	mt-Tl1	1	mito tsRNA
ID1305	AAGAAAGATTGCAAGAACTGCTAATTCATGCTTA	mt-Ts2	1	mito tsRNA
ID1828	CACTATGAAGCTAAGAGCGTTAACCTTTTA	mt-Tk	1	mito tsRNA
ID1849	TAGATTGAAGCCAGTAATAGGGTATTTAGC	mt-Tn	1	mito tsRNA
ID1883	AAGATATTAGTAAAATCAATTACATAACT	mt-Td	1	mito tsRNA
ID2300	ACCTTGTTCCCAGAGGTTCAAATCCTCTCCA	mt-Tl1	1	mito tsRNA
ID2597	TAGGAACCAAAAACCTTGGTGCAAATCCAAA	mt-Tl2	1	mito tsRNA
ID1675	TCAAAGTTAAATTATAGATCAATAATCTATATATCTTA	mt-Td	0.99	mito tsRNA
ID1791	GTCTTGATAGTATAAACATTACTCTGGTCT	mt-Tt	0.99	mito tsRNA
ID1829	TAGATTGAAGCCAGTAATAGGGTATTT	mt-Tn	0.99	mito tsRNA
ID849	CACTATGAAGCTAAGAGCGTTAACCTTTTAAGTTAA	mt-Tk	0.98	mito tsRNA
ID967	AAGATATTAGTAAAATCAATTACATAACTTTGTA	mt-Td	0.98	mito tsRNA
ID1281	TTAACTAAATTTTCGTAGGTTTAATTCCTGCCAATA	mt-Tn	0.98	mito tsRNA
ID1636	CTAATTCATGCTTCCATGTTTAAAAACATGGCTTTCTT	mt-Ts2	0.98	mito tsRNA
ID2150	CACTATGAAGCTAAGAGCATTAACCTTTTAAG	mt-Tk	0.98	mito tsRNA
ID2176	CACTATGATGCTAAGAGCGTTAACCTTTTAA	mt-Tk	0.98	mito tsRNA

ID135	TAGGATAAGGTGTTT	mt-Tq	0.97	mito tsRNA
ID202	TGGTAATTAGTTTAAAAAAAAA	mt-Tr	0.97	mito tsRNA
ID871	CATAGTGTAGCTTAATATTAAAGCATCTGGCCTA	mt-Tv	0.97	mito tsRNA
ID1268	AAAGTTAAATTATAGATCAATAATCTATATATA	mt-Td	0.97	mito tsRNA
ID1275	TCAAAGTTAAATTATAGATCAATAATCTATATATCA	mt-Td	0.97	mito tsRNA
ID1336	TCAAAGTTAAATTATAGATCAATAATCTATATATA	mt-Td	0.97	mito tsRNA
ID1429	TCAAAGTTAAATTATAGATCAATAATCTATATATCTA	mt-Td	0.97	mito tsRNA
ID1433	CAAAGTTAAATTATAGATCAATAATCTATATATA	mt-Td	0.97	mito tsRNA
ID1513	CATTCAATAGATGTAGGATGAAGTCTTACAGTCCTT	mt-Ta	0.97	mito tsRNA
ID1646	TGAATCTGACAACAGGAAATAAACCTCCTTATTA	mt-Th	0.97	mito tsRNA
ID2107	CACTATGAGGCTAAGAGCGTTAACCTTTTAA	mt-Tk	0.97	mito tsRNA
ID2115	GGTAAAATGGCTGAGTAAGCATTAGACTGTAA	mt-Ty	0.97	mito tsRNA
ID2225	CTTGTTCCCAGAGGTTCAAATCCTCTCCA	mt-Tl1	0.97	mito tsRNA
ID163	AAGAAAGATTGCAAGA	mt-Ts2	0.96	mito tsRNA
ID937	GTTAATGTAGCTTAATAACAAAGCAAAGCACTGAAA	mt-Tf	0.96	mito tsRNA
ID1092	AGAAGTTTAGGATATACTAGTCCGCGAGCCTTCA	mt-Tw	0.96	mito tsRNA
ID1169	AGGGTATTTAGCTGTTAACTAAATTTTCGTAGGTTT	mt-Tn	0.96	mito tsRNA
ID1327	AAACCTTGTTCCCAGAGGTTCAAATCCTCTCCCA	mt-Tl1	0.96	mito tsRNA
ID1356	CACTATGATGCTAAGAGCGTTAACCTTTTAAGTTAA	mt-Tk	0.96	mito tsRNA
ID1459	GATAGAGTAAATTATAGAGGTTCAAGCCCTCTTATTTA	mt-Ti	0.96	mito tsRNA
ID1606	TAAATCTAAACACAGAGGTTTAAATCCTCTTTTTACC	mt-Ty	0.96	mito tsRNA
ID1617	TTAAGCTATCGGGCCCATACCCCGAAAACGTTGGTTTAAA	mt-Tm	0.96	mito tsRNA
ID1801	CACTATGAAGCTAAGAGCGTTAACCTTT	mt-Tk	0.96	mito tsRNA
ID2208	GTTCCCAGAGGTTCAAATCCTCTCCCTAATA	mt-Tl1	0.96	mito tsRNA
ID2231	CTTGTTCCCAGAGGTTCAAATCCTCTCCCA	mt-Tl1	0.96	mito tsRNA

ID2627	TTAAAGCAATTGATTTGCATTCAATAGATGTAGGATGAAG	mt-Ta	0.96	mito tsRNA
ID91	TGAAGCGTTCCATATTTTT	Gm24205	1	snsRNA
ID862	TTTGTGGTAGTGGGGGACTGCGTTCGCGCTCTCCCCTG	Gm22634	1	snsRNA
ID864	TGGTAGTGGGGGACTGCGTTCGCGCTCTCCCCTG	Rnu1b1	1	snsRNA
ID876	GTGGTAGTGGGGGACTGCGTTCGCGCTCTCCCCTG	Gm26444	1	snsRNA
ID1763	TTTTTGAGGCCTTGTCTTGACAAGGCT	Rnu5g	1	snsRNA
ID1764	AATTTTTTGAGGCCTTGTCTTGACAAGGCT	Rnu5g	1	snsRNA
ID1779	AATTTTTTGAGGCCTTGTTTCGGCAAGGCT	Gm25313	1	snsRNA
ID1842	AATTTTTTGAGGTCCTGCTCGTGCAGGGCT	Gm25099	1	snsRNA
ID1870	TTTTGAGGTCCTGCTCGTGCAGGGCT	Gm25099	1	snsRNA
ID1871	GTGGGGGACTGCGTTCGCGCTCTCCCCTG	Rnu1a1	1	snsRNA
ID2286	TAAAATTGGAACGATACAGAGAAGA	Gm22307	1	snsRNA
ID705	AAATGGATTTTTGGAACTAGGAG	Gm25939	0.99	snsRNA
ID1758	TGCGGGAAACTCGACTGCATAATTTGTGGTAGTGGGGGAC	Gm25189	0.99	snsRNA
ID2640	CCGGATGTGCTGACCCCTGCGATTTCCCC	Gm26444	0.99	snsRNA
ID683	AAATGGATTTTTGGAAGTAGGAG	Gm25202	0.98	snsRNA
ID798	AAAGATTTCCGTGGAGAGGAAC	Gm23143	0.98	snsRNA
ID1036	ATACTTACCTGGCAGGGGAGATACCATGATCACGAAGG	Gm22513	0.98	snsRNA
ID1523	TGGTTTCTCTTCAGATCGTATAAATCTTTCGCC	Rnu5g	0.98	snsRNA
ID1754	TATACTAAAATTGGAACGATACAGAGAAGATTAGCA	Gm24705	0.98	snsRNA
ID2172	TACTAAAATTGGAACGATACAGAGAAGA	Gm23244	0.98	snsRNA
ID2526	TTTTACTAAAGATTTCCGTGGAGAGGAACAAC	Gm25313	0.98	snsRNA
ID2598	TGCATAATTTGTGGTAGTGGGGGACTGCG	Gm24924	0.98	snsRNA
ID2649	TGCATAATTTGTGGTAGTGGGGGAC	Gm22317	0.98	snsRNA
ID2750	CGGATGTGCTGACCCCTGCGA	Gm22068	0.98	snsRNA

ID1485	TCGACTGCATAATTTGTGGTAGTGGGGGACTGCG	Gm22614	0.97	snsRNA
ID1574	TTAGCATGGCCCCTGCGCAAGGATGACACGCAAATTCG	Gm24389	0.97	snsRNA
ID2050	ACTCCGGATGTGCTGACCCCTGCGATTTCCCC	Gm23238	0.97	snsRNA
ID2511	AGTCGGCATTGGCAATTTTTGACAGTCTCT	Gm25899	0.97	snsRNA
ID2743	TCTTAAACCAATTTTTTGAGGCCTTGTCT	Rnu5g	0.97	snsRNA
ID37	ACCTCCAGGAACGGTGCACCA	Gm23849	0.96	snsRNA
ID1607	TATATTAAATGGATTTTTGGAAGTAGGAGTTGGAA	Gm25813	0.96	snsRNA
ID2292	TGCTGACCCCTGCGATTTCCCCAAA	Rnu1a1	0.96	snsRNA
ID2541	GCATAATTTGTGGTAGTGGGGGAC	Gm25890	0.96	snsRNA
ID2557	TCTTACACTAATTTTTTGAGGCCTTGCT	Gm24871	0.96	snsRNA
ID1	TGGAGTGTGACAATGGTGTTTGA	Gm29966	1	lncsRNA
ID129	AACGCCATTATCACACTAAATAT	Gm29966	1	lncsRNA
ID1249	AGCTCGCTCTGAAGGCCTGTTTCCTAGGCTACA	Rmrp	1	lncsRNA
ID1813	AATTTTTTGAGGCCTTGCTTTAGCAAGGCT	B930036N10Rik	1	lncsRNA
ID1827	TGGAGTGTGACAATGGTGTTTGTT	Gm29966	1	lncsRNA
ID1906	AATTTTTTGAGGCCTTGCTTTAGCAAGGCTA	B930036N10Rik	1	lncsRNA
ID2744	TCTTTGGTTATCTAGCTGTATGAA	C130071C03Rik	1	lncsRNA
ID2758	TACTGAGAAAAACCTTTTTTATCCAAGAGA	Gm21221	1	lncsRNA
ID2780	TGGAATGTAAGGAAGTGTGTGT	Gm28653	1	lncsRNA
ID2781	TAAGGCACGCGGTGAATGCCAAT	Mir124a-1hg	1	lncsRNA
ID2789	TAAGGCACGCGGTGAATGCA	Mir124a-1hg	1	lncsRNA
ID2797	AGCAACAACATAAACTGCGAAACAGGTGA	4933440M02Rik	1	lncsRNA
ID2798	TACCAATTGAGCTATATCCCAACCTCCATT	Gm10010	1	lncsRNA
ID2799	TAAATGGACTTTGGCCCCAGGTCAATTCTGGC	Gm10619	1	lncsRNA
ID2800	TCAATAAATAGAGGCTAGCATATGCCAGGA	Gm10619	1	lncsRNA

ID2804	CTCAGAACTAGTGTATATTTGGCCAAAAGT	1700001L05Rik	1	lncsRNA
ID2806	TGAAAGGAACTTTCCTAGAGTTATGCACTGT	Gm5878	1	lncsRNA
ID2808	TAACTCTAAATGGACTTTGGCCCCAGGTC	Gm10619	1	lncsRNA
ID2809	TTGGTCTTCTGGTTGTGATAGTTTTTCTCCC	4930444F02Rik	1	lncsRNA
ID2812	TGTGAAGAAAGCCGATGGAATTCAGGGA	1700008K24Rik	1	lncsRNA
ID2814	CTTCTTGATAAGAAAAATGCTGCCTAGATA	4930444F02Rik	1	lncsRNA
ID2815	TAAGAGAATTCAGTGTTGCTCTGAGGACA	Gm5878	1	lncsRNA
ID2818	TTATCTCTGTGATCTAACTGATGCCGTGGC	1700009C05Rik	1	lncsRNA
ID2821	TGATACATGGTTAGTGTGAATTTCTAGGA	1700009C05Rik	1	lncsRNA
ID2823	TAGAAACTGCCTGGTGCAGATGCCATGGTGT	1700042G15Rik	1	lncsRNA
ID2824	TAAGTTGGTTGTTGTTCTGTCATGCACTGT	1700009C05Rik	1	lncsRNA
ID2828	GAAATGAATGAGGACCTGGTCCTGTGGGT	Gm5878	1	lncsRNA
ID2837	TTCAAGGTCAAAGTCAGAAACCACTCAGATT	Gm30132	1	lncsRNA
ID2840	TCAGAGAACAAGTTCACTCTGGCTGGAAGT	4933440M02Rik	1	lncsRNA
ID2843	TAAGATGATTGGAATTTTGGTTTTTCCGAT	1700008K24Rik	1	lncsRNA
ID2850	GTCCGTACCAGGTAGAGACCAGATCCAATGT	4933440M02Rik	1	lncsRNA
ID2862	TAGAAACTGCCTGGTGCAGATGCCATGGT	1700042G15Rik	1	lncsRNA
ID2863	TAGAAGACGAGTTTGGACTGAGCGAAATTC	Gm5878	1	lncsRNA
ID2864	TGAATCATCTACTACACCCTGTGGAAACAAT	Cep83os	1	lncsRNA
ID2865	TTTCACAGATGAGGAAACTGAAGCTTAGGT	1700001L05Rik	1	lncsRNA
ID2866	TGAAATGAATGAGGACCTGGTCCTGTGGGT	Gm5878	1	lncsRNA
ID2873	TAGAGTCTCTCCATCCAATTTAGGAATTCTGT	1700020N18Rik	1	lncsRNA
ID2880	AACTTGACTACATCTGGAATTAACTAAAACCC	Gm5535	1	lncsRNA
ID2882	TACCATAGAAGACGAGTTTGGACTGAGCGT	Gm5878	1	lncsRNA
ID2883	TTGCCACCGAGATTCGAGTGAAGAATGAGA	1700042G15Rik	1	lncsRNA

ID2902	AGCAACAACATAAACTGCGAAACAGGTGAAGT	4933440M02Rik	1	lncsRNA
ID2911	TGATACATGGTTAGTGTGAATTTCTAGGAAGT	1700009C05Rik	1	lncsRNA
ID2917	ATGAAATGAATGAGGACCTGGTCCTGTGGGT	Gm5878	1	lncsRNA
ID3008	TAAGGCACGCGGTGAATGCCAAAA	Mir124a-1hg	1	lncsRNA
ID1202	TTCGGCTGATGATTGCTGTTGAGACTTGGAAATCTGATT	Snhg1	0.98	lncsRNA
ID1711	CCACGAGGACGAGACGTAGCGTTCCCTCCTGAGCG	Rnu3b3	1	snosRNA
ID2356	TTGGTAGTGGTGAGCCTATGGTTTTCTGAAG	Snord65	1	snosRNA
ID2398	TCGCTGTGATGATGGATTCCAAAACCATTCG	Snord14d	1	snosRNA
ID2793	ACATAAAATTCATGCTCAATAGGATTACGCTGAGGCCC	Gm26434	1	snosRNA
ID2795	TAAAATTCATGCTCAATAGGATTACGCTGAGGCCC	Gm22524	1	snosRNA
ID2859	AATAGGATTACGCTGAGGCCC	Gm23560	1	snosRNA
ID3011	AGGATTACGCTGAGGCCC	Gm26499	1	snosRNA
ID3016	ATAAAATTCATGCTCAATAGGATTACGCTGAGGCCC	Gm25988	1	snosRNA
ID3017	AAAATTCATGCTCAATAGGATTACGCTGAGGCCC	Gm22627	1	snosRNA
ID3020	GGTCAATGATGACAACCCAATGTCATGAAGAAAGGT	Gm26392	1	snosRNA
ID3023	GGTCAATGATGACAACCCAATGTCAT	Gm22252	1	snosRNA
ID2381	TGCAAGGACTTGTCATAGTTACACTGACT	Gm26330	0.99	snosRNA
ID455	AAGACTATACTTTCAGGATCA	Rnu3b2	0.98	snosRNA
